# On specimen killing in the era of conservation crisis – A quantitative case for modernizing taxonomy and biodiversity inventories

**DOI:** 10.1371/journal.pone.0183903

**Published:** 2017-09-13

**Authors:** Patrick O. Waeber, Charlie J. Gardner, Wilson R. Lourenço, Lucienne Wilmé

**Affiliations:** 1 ETH Zurich, Department of ITES, Forest Management and Development (ForDev) Group, Universitätstrasse 16, Zurich, Switzerland; 2 Durrell Institute of Conservation and Ecology (DICE), University of Kent, Canterbury, United Kingdom; 3 Muséum national d’Histoire naturelle, Sorbonne Universités, Institut de Systématique, Evolution, Biodiversité (ISYEB), UMR7205-CNRS, MNHN, UPMC, EPHE, CP 53, 57 rue Cuvier, Paris, France; 4 Missouri Botanical Garden, Madagascar Research & Conservation Program, BP, Antananarivo 101, Madagascar; 5 World Resources Institute, Washington, D.C., United States of America; Tierarztliche Hochschule Hannover, GERMANY

## Abstract

**Background to the work:**

For centuries taxonomy has relied on dead animal specimens, a practice that persists today despite the emergence of innovative biodiversity assessment methods. Taxonomists and conservationists are engaged in vigorous discussions over the necessity of killing animals for specimen sampling, but quantitative data on taxonomic trends and specimen sampling over time, which could inform these debates, are lacking.

**Methods:**

We interrogated a long-term research database documenting 2,723 land vertebrate and 419 invertebrate taxa from Madagascar, and their associated specimens conserved in the major natural history museums. We further compared specimen collection and species description rates for the birds, mammals and scorpions over the last two centuries, to identify trends and links to taxon descriptions.

**Results:**

We located 15,364 specimens documenting endemic mammals and 11,666 specimens documenting endemic birds collected between 1820 and 2010. Most specimens were collected at the time of the *Mission Zoologique Franco-Anglo-Américaine* (MZFAA) in the 1930s and during the last two decades, with major differences according to the groups considered. The small mammal and bat collections date primarily from recent years, and are paralleled by the description of new species. Lemur specimens were collected during the MZFAA but the descriptions of new taxa are recent, with the type series limited to non-killed specimens. Bird specimens, particularly of non-passerines, are mainly from the time of the MZFAA. The passerines have also been intensely collected during the last two decades; the new material has been used to solve the phylogeny of the groups and only two new endemic taxa of passerine birds have been described over the last two decades.

**Conclusions:**

Our data show that specimen collection has been critical for advancing our understanding of the taxonomy of Madagascar’s biodiversity at the onset of zoological work in Madagascar, but less so in recent decades. It is crucial to look for alternatives to avoid killing animals in the name of documenting life, and encourage all efforts to share the information attached to historical and recent collections held in natural history museums. In times of conservation crisis and the advancement in digital technologies and open source sharing, it seems obsolete to kill animals in well-known taxonomic groups for the sake of enriching natural history collections around the world.

## Introduction

Collections of animal specimens have formed the basis of taxonomic work since the first attempts to document the richness of life on Earth. However, although the International Code of Zoological Nomenclature (ICZN) recommends the designation of a single specimen as holotype (article 16.4.1 of the ICZN, http://www.iczn.org/iczn/index.jsp) for the description of a new species or subspecies published after 1999, a preserved specimen has never been mandatory [1]. After centuries of specimen collection, we now have a fair understanding of the taxonomy of some animal groups, including in the tropics [e.g., 2–5]. This raises the question of whether further specimen collection within these groups is still required, particularly in cases where it may have negative impacts for the species. Recently, Minteer and colleagues [6] highlighted the fact that the collection of voucher specimens can potentially exacerbate the conservation status of a species or contribute to its extinction, as has occurred with the Great Auk (*Pinguinus impennis*; [7]) and Socorro Elf Owl (*Micrathene whitneyi soccorroensis*; [8]). Minteer and colleagues’ opinion paper revived a long-lasting debate between those who defend the value of collections for taxonomy or other scientific purposes [9–15], and researchers advocating for alternative and innovating means for documenting life [16,17]. However, there remains a lack of empirical data to underpin these arguments from both sides.

The purpose of this paper is to contribute to the ongoing debate about the usefulness of systematic collections to describe new taxa or to document biodiversity. Hence, we present a temporal analysis of specimens housed in natural history museums, mainly in Europe and North America, with large collections made in 1929–1931 and over the last two decades in Madagascar. We have specifically looked at two well-known groups of vertebrates, birds and mammals; and to a group of invertebrates for which the preservation of specimens is usually needed to describe new taxa. Our analysis aims to identify ancient and recent trends as well as their putative causality.

## Results

**Fig 1:**
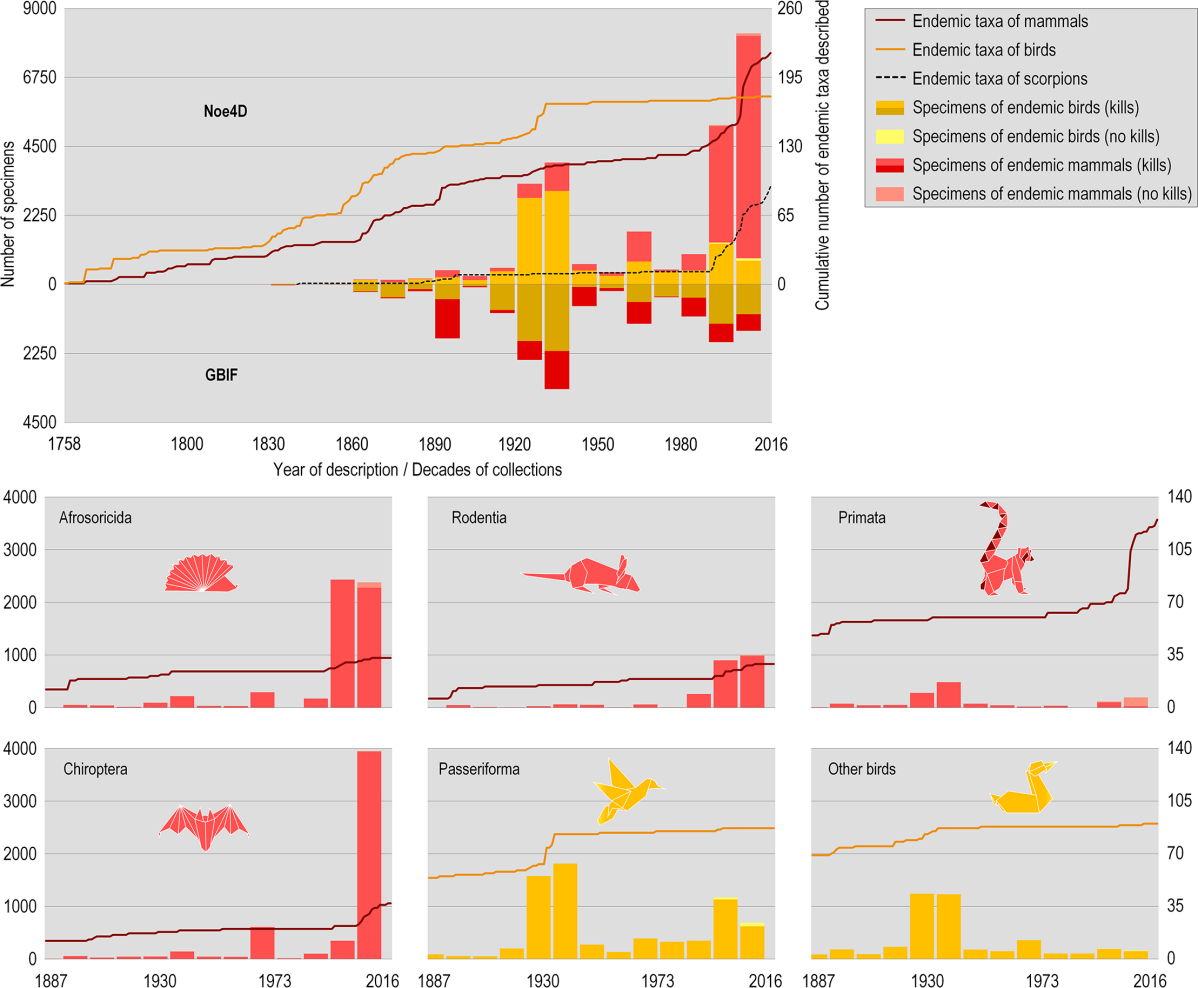
Description of endemic species and subspecies of birds, mammals, and scorpions over time, and number of specimens of endemic birds and mammals. The details of scorpion taxa described since the 1990s are presented in supporting information (S1 Case, S2 Table). Specimens and taxa are based on Noe4D (upper portion of top left graph) and GBIF (lower portion). Overall, the grand majority of specimens consists of killed specimens, with the non-killed specimens gaining momentum in the last two decades. Since the 1930s only a few new bird taxa have been described, while the description of mammals has surged since the mid-1990s with the description of new species of small mammals in the orders Afrosoricida and Rodentia, and the application of the Phylogenetic Species Concept and new molecular tools to the systematics of lemurs [24,25]. Since the 1990s, thousands of birds and mammals have been removed from the wild to document the diversity of these two groups in Madagascar. Despite increasing efforts to document the other endemic vertebrates since the 1990s the only class for which we have a good taxonomic knowledge in Madagascar, and have done for over 50 years, is the birds. In total, there have been 92 endemic taxa (species and subspecies) of scorpions, 176 endemic taxa of birds and 219 endemic taxa of mammals described by the end of 2016.

Through the Noe4D database, we have identified 13,250 specimens (killed and non-killed) of birds (11,666 or 88.0% endemics) and 18,341 specimens of mammals (15,364 or 83.8% endemics). These have been collected between 1820 and 2010 in Madagascar. They are housed in the collections of the major natural history museums, e.g., American Museum of Natural History, New York, the Field Museum of Natural History, Chicago, IL, the Musée national d'Histoire naturelle, Paris, or The Museum of Texas Tech University, Lubbock, TX (Fig 1, S1 Table).

The Global Biodiversity Information Facility (GBIF http://www.gbif.org) database has confirmed the same trends as the Noe4D (Fig 1); a first major peak during the *Mission Zoologique Franco-Anglo-Américaine* (MZFAA), two minor peaks in the 1960s and 1980s and an increase over the last two decades. The main discrepancies are within the Class of mammals, the most evident ones in the 1890s (GBIF) and during the last two decades (Noe4D).

Scorpions are collected by overturning rocks or the use of ultra-violet light, as well as extraction methods such as Berlese traps, Winkler traps, and (more recently) pitfall traps. Pitfall traps have been deployed by several multidisciplinary teams over the last two decades to inventory terrestrial small mammals, reptiles and amphibians [e.g., 18,19]. Some invertebrates are often caught in the pitfalls, including thousands of scorpions [20]. Pitfall traps per se are not selective and are not intended to kill animals [e.g., 21].

### The two main collection periods

The collections of endemic birds show two periods of intensive collection, with a major peak during the MZFAA between April 1929 and May 1931, during which the birds were shot with guns or brought in by villagers [22]. The second peak, spanning the last two decades, is mainly focused on passerines (Fig 1). Over this period, collection of bird specimens has been carried out primarily as part of site-based assessments of biodiversity, and the birds were mainly trapped by means of mist-nets, which explains the high number of passerines. The endemic terrestrial birds are mainly non-passerines [23]; these are difficult to catch in mist-nets and are hence less common in recent collections given that mist-netting is the main methodology applied in forests for inventory of the avifauna (e.g., from 1990s onwards). For example, the genus *Mentocrex* is currently recognized by three endemic taxa (two endemic species including a monotypic species and a species with two subspecies, or one endemic species with three subspecies). We have located some 65 specimens for these taxa including 51 (78%) shot during the time of the MZFAA. A similar pattern is seen amongst the endemic birds of prey in the family Accipitridae (Accipitrifomes), the pigeons (Columbidae, Columbiformes) and the sandgrouse (Pteroclididae, Pterocliformes), for which a total of 299 out of 446 (67%), 224/348 (64%), and 33/45 (73%) specimens, respectively, were collected during the time of the MZFAA expedition. Specimens of endemic non-passerine birds were almost as numerous or more numerous than passerine birds until the decade ending 1970, but have represented less than a quarter of the endemic bird specimens subsequently (Table 1).

**Table 1:** Specimens of endemic mammals and birds documented in Noe4D and number of endemic species and subspecies described for decades starting in 1920. (Color code as in Fig 1; darker color for specimens with killed animals; lighter color for specimens without killed animals; number of taxa for species and subspecies recognized today in non colored lines in the form of ‘number of taxa with type material based on killed animals’ / ‘number of taxa with type material excluding killed animals’–excluding the taxa described and put in synonymy).

Order	1920–1930	1930–1940	1940–1950	1950–1960	1960–1970	1970–1980	1980–1990	1990–2000	2000–2016
Anseriformes	89	20	3	6	1	1	-	2	-
	**-**	**-**	**-**	**-**	**-**	**-**	**-**	**-**	**-**
Galliformes	31	17	3	1	4	-	1	5	-
	**-**	**-**	**-**	**-**	**-**	**-**	**-**	**-**	**-**
Podicipediformes	34	15	5	5	8	-	-	2	-
	**-**	**1**	**-**	**-**	**-**	**-**	**-**	**-**	**-**
Columbiformes	121	103	15	4	13	10	12	20	18
	**-**	**1**	**-**	**-**	**-**	**-**	**-**	**-**	**-**
Mesitornithiformes	53	28	7	4	13	1	1	2	-
	**-**	**-**	**-**	**-**	**-**	**-**	**-**	**-**	**-**
Pterocliformes	24	9	1	6	4	-	-	-	-
	**-**	**-**	**-**	**-**	**-**	**-**	**-**	**-**	**-**
Cuculiformes	116	151	42	19	114	21	18	27	7
	**-**	**1**	**-**	**1**	**-**	**-**	**-**	**-**	**-**
Caprimulgiformes	88	162	9	6	21	6	14	13	17
	**-**	**-**	**-**	**-**	**-**	**-**	**-**	**-**	**-**
Charadriiformes	63	52	13	8	11	6	-	1	-
	**-**	**-**	**-**	**-**	**-**	**-**	**-**	**-**	**1**
Gruiformes	111	144	25	13	26	9	13	7	12
	**1**	**1**	**-**	**-**	**-**	**-**	**-**	**-**	**1**
Pelecaniformes	18	58	6	12	12	5	4	3	-
	**1**	**-**	**-**	**-**	**-**	**-**	**-**	**-**	**-**
Accipitriformes	152	141	11	8	25	11	1	9	5
	**-**	**-**	**-**	**-**	**-**	**-**	**-**	**-**	**-**
Strigiformes	49	42	5	5	9	1	3	23	14
	**1**	**-**	**-**	**-**	**-**	**-**	**-**	**-**	**-**
Coraciiformes	173	185	14	20	49	22	23	65	73
	**-**	**-**	**-**	**-**	**-**	**-**	**-**	**-**	**-**
Leptosomiformes	30	35	3	5	11	3	1	-	-
	**-**	**-**	**-**	**-**	**-**	**-**	**-**	**-**	**-**
Psittaciformes	48	45	10	22	26	5	3	11	2
	**2**	**-**	**-**	**-**	**-**	**-**	**-**	**-**	**-**
Falconiformes	41	23	7	3	10	2	-	1	1
	**-**	**-**	**-**	**-**	**-**	**-**	**-**	**-**	**-**
Sub-total non-Passeriformes	1241	1230	179	147	357	103	103	191	149
	**5**	**4**	**-**	**1**	**-**	**-**	**-**	**-**	**2**
Passeriformes	1577	1813	273	134	390	326	349	1132	621
	-	-	-	-	-	-	-	37	69
	**5**	**20**	**-**	**1**	**-**	**1**	**-**	**2**	**-**
Sub-total birds	2818	3043	452	281	747	429	452	1323	770
	-	-	-	-	-	-	-	37	69
	**10**	**24**	**-**	**2**	**-**	**1**	**-**	**2**	**2**
Afrosoricida	90	217	31	27	291	-	171	2433	2279
	-	-	-	-	-	-	-	-	100
	**1**	**3**	**-**	**-**	**-**	**-**	**-**	**6**	**3**
Primata	277	483	74	43	19	35	2	96	25
	1	-	-	-	-	-	-	19	167
	**-**	**2**	**-**	**-**	**-**	**3**	**3**	** 4**	**24 / 21**
Chiroptera	45	141	42	39	609	13	101	344	3942
	-	-	-	-	-	-	-	-	111
	**1**	**1**	**-**	**1**	**-**	**-**	**-**	**2**	**11**
Carnivora	24	30	14	-	3	1	1	21	25
	-	-	-	-	-	-	-	-	1
	**1**	**-**	**1**	**-**	**-**	**1**	**-**	**-**	**-**
Rodentia	26	61	51	6	57	4	259	898	978
	-	-	-	-	-	-	-	-	21
	**1**	**-**	**2**	**1**	**1**	**-**	**-**	**5**	**5**
Sub-total mammals	462	932	212	115	979	53	534	3792	7249
	1	-	-	-	-	-	-	19	400
	**4**	**6**	**3**	**2**	**1**	**4**	**4**	**17**	**43 / 21**

Specimen collection trends for the endemic land mammals vary between orders. The endemic carnivores (Eupleridae) are represented by 16 endemic species and subspecies and are documented by 156 specimens in the Noe4D database, including 54 collected at the time of the MZFAA and 46 (45 kills) during the last two decades (Table 1). The endemic micro-mammals of Madagascar belong to the Tenrecidae in the Order Afrosoricida and the rodents; they are represented by 33 and 29 endemic species and subspecies, and documented by 5754 and 2414 specimens respectively in Noe4D (Fig 1). Most micro-mammal specimens were collected during the last two decades, and led to the description of nine tenrec (27.3%) and 10 rodent (34.5%) taxa during this period. The Chiroptera (bats) have been massively collected during the last two decades (Table 1), leading to the description of 15 species out of a total number of 37 taxa documented by 3942 specimens (Fig 1). Bats are usually trapped by mist-nests or harp traps, often deployed near a roost [26], which explains the large number of specimens obtained.

### Phylogenetic and taxonomic considerations based on new technologies

Most of the birds formerly known as Malagasy greenbuls, previously placed within the Pycnonotidae, as well as several taxa previously considered amongst the sylviids or timaliids, have been recently recognized as an endemic lineage, the family Bernieridae [27]. The study was based on molecular material of birds killed and preserved as museum specimens after blood or tissue samples have been extracted. Some 970 specimens have been located for the 15 taxa within the Bernieridae, including 289 (30%) collected during the time of the MZFAA and 396 (41%) during the last two decades (S3 Table). All of the birds for which blood or tissue samples have been collected over the last decades to allow the molecular analyses were killed and preserved as specimens.

Some specimens collected during the MZFAA have been included in the type material of recently described taxa, including the gull *Larus dominicanus melisandae* Jiguet 2002 (Paratypes MNHN 1932-161/2) [28], the rodent *Monticolomys koopmani* Carleton & Goodman 1996 [29], and, the carnivore *Galidictis grandidieri* Wozencraft 1986 (Paratype AMNH 100478) [30]. At least 85 specimens of the Least Concern *Monticolomys koopmani* (Holotype AMNH 100727) have been collected over the last two decades, and three specimens of *Galidictis grandidieri* were collected in Tsimanampetsotsa in 2002 and 2004 and exported to the USA, despite the species having an IUCN conservation status of Endangered since 1996 [31].

The primates (lemurs) were also intensively collected at the time of the MZFAA but modern collections are limited and mainly exclude dead animals (Table 1, Fig 1). In the Noe4D, we have located a total of 1440 specimens of lemurs including 785 that were shot during the time of the MZFAA. As many as 307 specimens have been collected during the last two decades, of which 121 involved killed specimens and 186 are limited to material excluding a dead animal for the description of 21 lemur taxa (Table 2). A killed specimen is an animal killed and prepared for preservation for scientific purposes in a zoological collection; it can be mounted, turned into a skin, preserved in spirit, or a skeletal or skull preserved separately. Since 1988, only nine lemur species have been described with associated killed museum specimens, and several species have been described with the type specimens kept in captivity (*Hapalemur aureus* Meyer, Albignac, Peyrieras, Rumpler, Wright 1987, *Microcebus jollyae*, *M*. *mittermeieri* and *M*. *simmonsi* Louis Jr., Coles, Andriantompohavana, Sommer, Engberg, Zaonarivelo, Mayor, Brenneman 2006). *Mirza zaza* was described in 2005 on the basis of a small piece of ear skin collected from a life individual, together with old specimens collected from the same locality in the late 19^th^ century and housed at the National Museum of Natural History in Leiden, NL [32]. In addition, as many as 24 taxa of lemurs have been described since 2005 based on ear clips, blood samples and other material but without a killed specimen. The type material of *Avahi cleesei* (AIMZ #13854) does not include any killed specimens, instead relying on hair, photos, video, and audio recordings [33] (Table 2).

**Table 2:** Recent lemur taxa described without killing or removing animals from the wild into captivity.

Name	Descriptor
*Avahi betsileo*	Andriantompohavana, Lei, Zaonarivelo, Engberg, Nalanirina, McGuire, Shore, Andrianasolo, Herrington, Brenneman, Louis Jr. 2007 [34]
*Avahi cleesei*	Thalmann, Geissmann 2005 [33]
*Avahi mooreorum*	Lei, Engberg, Andriantompohavana, McGuire, Mittermeier, Zaonarivelo, Brenneman, Louis Jr. 2008 [35]
*Cheirogaleus andysabini*	Lei, McLain, Frasier, Taylor, Bailey, Engberg, Ginter, Nash, Randriamampionona, Groves, Mittermeier, Louis Jr. 2015 [36]
*Cheirogaleus lavasoensis*	Thiele, Razafimahatratra, Hapke 2013 [37]
*Cheirogaleus shethi*	Frasier, Lei, McLain, Taylor, Bailey, Ginter, Nash, Randriamampionona, Groves, Mittermeier, Louis Jr 2016 [38]
*Lepilemur ahmansonorum*	Louis Jr., Coles, Andriantompohavana, Sommer, Engberg, Zaonarivelo, Mayor, Brenneman 2006 [39]
*Lepilemur betsileo*	Louis Jr., Coles, Andriantompohavana, Sommer, Engberg, Zaonarivelo, Mayor, Brenneman 2006 [39]
*Lepilemur fleuretae*	Louis Jr., Coles, Andriantompohavana, Sommer, Engberg, Zaonarivelo, Mayor, Brenneman 2006 [39]
*Lepilemur grewcockorum*	Louis Jr., Coles, Andriantompohavana, Sommer, Engberg, Zaonarivelo, Mayor, Brenneman 2006 [39]
*Lepilemur hubbardorum*	Louis Jr., Coles, Andriantompohavana, Sommer, Engberg, Zaonarivelo, Mayor, Brenneman 2006 [39]
*Lepilemur jamesorum*	Louis Jr., Coles, Andriantompohavana, Sommer, Engberg, Zaonarivelo, Mayor, Brenneman 2006 [39]
*Lepilemur milanoii*	Louis Jr., Coles, Andriantompohavana, Sommer, Engberg, Zaonarivelo, Mayor, Brenneman 2006 [39]
*Lepilemur petteri*	Louis Jr., Coles, Andriantompohavana, Sommer, Engberg, Zaonarivelo, Mayor, Brenneman 2006 [39]
*Lepilemur scottorum*	Lei, Engberg, Andriantompohavana, McGuire, Mittermeier, Zaonarivelo, Brenneman, Louis Jr. 2008 [34]
*Lepilemur seali*	Louis Jr., Coles, Andriantompohavana, Sommer, Engberg, Zaonarivelo, Mayor, Brenneman 2006 [39]
*Lepilemur tymerlachsoni*	Louis Jr., Coles, Andriantompohavana, Sommer, Engberg, Zaonarivelo, Mayor, Brenneman 2006 [39]
*Lepilemur wrightae*	Louis Jr., Coles, Andriantompohavana, Sommer, Engberg, Zaonarivelo, Mayor, Brenneman 2006 [39]
*Microcebus ganzhorni*	Hotaling, Foley, Lawrence, Bocanegra, Blanco, Rasoloarison, Kappeler, Barrett, Yoder, Weisrock 2016 [40]
*Microcebus lehilahytsara*	Roos, Kappeler 2005 [32]
*Microcebus macarthurii*	Radespiel, Olivieri, Rasolofoson, Rakotondratsimba, Rakotonirainy, Rasoloharijaona, Randrianambinina, Ratsimbazafy, Ratelolahy, Randriamboavonjy, Rasolofoharivelo, Craul, Rakotozafy, Randrianarison 2008 [41]

## Discussion

In the last decades of the 19^th^ century, peaks in mammal collections are attributed to the initiative of single researchers such as Charles I. Forsyth-Major who collected small mammals (Afrosoricida & Rodentia) between 1894 and 1896 for the British Museum of Natural History (S1 Fig) [42]. Many of these specimens have not been included in the Noe4D database but are included in GBIF (Fig 1). After the MZFAA, the crisis decades pre-and post WW II brought a slowdown in zoological expeditions. After independence in 1960, Madagascar became attractive for mainly French and US based zoologists (e.g., Charles Domergue, Rose Lavite, Randolph L. Peterson, Philippe Milon). In the late 1980s and onwards, international attention for biodiversity concerns brought a lot of funding for conservation and research [43,44], and a lot of biodiversity inventories during which thousands of birds and mammals were killed, while remarkable few new taxa were described. These specimens are mainly housed at the Field Museum of Natural History in Chicago, and are therefore absent from GBIF which compiles primarily European collections. Both databases show gaps in collections until the 1880s; this is mainly explained by the fact that most historical specimens do not hold information on the locality or the date of collections, and are therefore less present in databases. The endemic passerine Helmet Vanga *Euryceros prevostii* belonging to the Vangidae bird family represents a classic example. The monotypic genus and the species have been described in 1831 by Lesson based on a specimen with no date and reported as “*originaire des Indes orientales*, *et très probablement des îles de Sumatra ou de Bornéo*.” (translated as “originating from Eastern India and highly likely from Sumatra or Borneo.” [45:pp422–423]).

The most recent described endemic species explosion is based on cryptic taxa of small mammals, bats and nocturnal lemurs (e.g., seven species in the genus *Microgale*, 19 taxa in the genus *Lepilemur*, 20 species in the genus *Microcebus*, six species in the genus *Miniopterus*). For these taxa morphometrics and external description are commonly insufficient to identify and describe the species. Hence, killing specimens is of little use for taxonomic purposes in some of these groups, particularly the nocturnal lemurs [e.g., 36,40]. The development of alternative methods to identify species limits and document biodiversity, including genetics, acoustics and photography allied with high quality blood, tissue and faecal material, now hold more potential information on species taxonomy, history, genetics, behaviour, parasite loads and other features than a killed specimen [e.g., 17,27,33]. Another advantage of such type material is related to their more efficient long-term storage and maintenance compared to the classic killed specimen, but see [46,47].

Taxonomy, systematics or natural history studies have relied on killing animals for centuries, and they still do nowadays despite innovative methods for biodiversity assessment [cf. 6,16,17]. In the case of the description of a new animal species, the International Code of Zoological Nomenclature does not require the killing of an animal [1]. Our study clearly shows that for some animal groups, such as larger mammals or ground living birds, a majority of descriptions were based on collecting killed animal specimens, a practice which had its peak in the 1930s. For other animal groups, the description of new species is still continuing and still requires the removal of animals from the wild. This has to do with the biology of the animal groups targeted and is the case for more elusive and smaller animals. For example, the discovery and description of new taxa of scorpions have increased with specific trapping features, such as pitfalls or extractive methods, allowing the capture of fossorial scorpions (but see S1 Case). The involvement of one specialist in an order or a family, together with the availability of material, can lead to a steep increase in the number of new taxa described, as in the case of the scorpions or the diplopods [48].

Populations or entire species are threatened [31] and recent estimates show that more than half of the Earth’s wildlife has been lost during the past 40 years [49,50]. Globally, 6.2% and 14.3% of birds and mammals are Endangered [31]. Madagascar in particular is facing a conservation crisis [44]. It seems counterintuitive to insist on the need to kill individuals to supply natural history collections around the world in a time of a global conservation crisis when more species are going extinct at a faster pace than ever [51]. Advances in digital and molecular technologies, as well as open source sharing, render such specimens more redundant. As a general rule for future taxonomic and biodiversity inventories, we propose that the new technologies may be considered as a baseline while killing specimens could be used for verification purposes ([e.g., 16,52], but see [13:p435] “modern descriptions shouldn’t be done without material evidence through at least one museum ‘type’ specimen, carrying many characters that cannot be seen on photographs”).

Natural history museums should invest in digitising their material to share every detail about specimens, including pictures or scans of the animals or parts of them. Many herbaria have begun a comprehensive effort to scan and digitise type specimens, and, more recently, all specimens of plants in Paris, but also in Leiden and New York, are being scanned and made available online [e.g., 53]. Similar efforts would facilitate the sharing and provision of information for animal specimens, reducing the need for further specimen collection. In order for collections to become more meaningful for research, it is suggested that updates on museum material becomes available online for taxonomic as well as biodiversity inventories [e.g., 54,55].

The collection of killed specimens can be justified in most instances, and remains needed. Killing an animal will likely not push any species towards extinction [11,56]. What can be harmful for a population, however, is the collection of large series (e.g., of invertebrates), as in the case of the use of pitfalls, where hundreds of animals can be trapped within a course of a few days. Some of the Malagasy scorpions may be considered threatened, both by the destruction of their environment, and by intensive collections. These can include not only academic activities, but also collections for the pet trade and amateur naturalist collectors. These species include some large taxa of the genus *Opisthacanthus*, but especially the elements of the endemic family Heteroscorpionidae, genus *Heteroscorpion*. One good example is *H*. *magnus*, a species endemic to the region of Daraina. These scorpions present extremely long biological cycles and the recovery of their populations from collections may require several decades [57,58]. Where we have a lack of data and an urgent need to describe and assess biodiversity before it goes extinct [59], as is the case of most groups of invertebrates, scientific rigor still relies on collections with killed specimens [but see 17, the preservation issue of the meiofauna best “conserved” with high quality photographs and movies]. With very few exceptions, no invertebrates are IUCN Red-listed and no threat assessments exist [31]. In the case of the birds of Madagascar, for which ample collections are available, mostly based on detailed collections during the MZFAA [22], and where endemics are threatened, such kills should be carefully considered and justified by specific studies. In particular, they should not be considered necessary for the purpose of site inventory, especially when a picture or other means can document the taxa without killing any individual. In light of the recurring debates over specimen collection, we strongly encourage researchers to be more careful with calling for specimens; there is no rule of thumb we can propose here, but before any specimen collection is envisioned, it should be assessed by whether there is truly a need and gain for doing so, and this depends on the groups of animals under consideration.

## Methods

**Fig 2:**
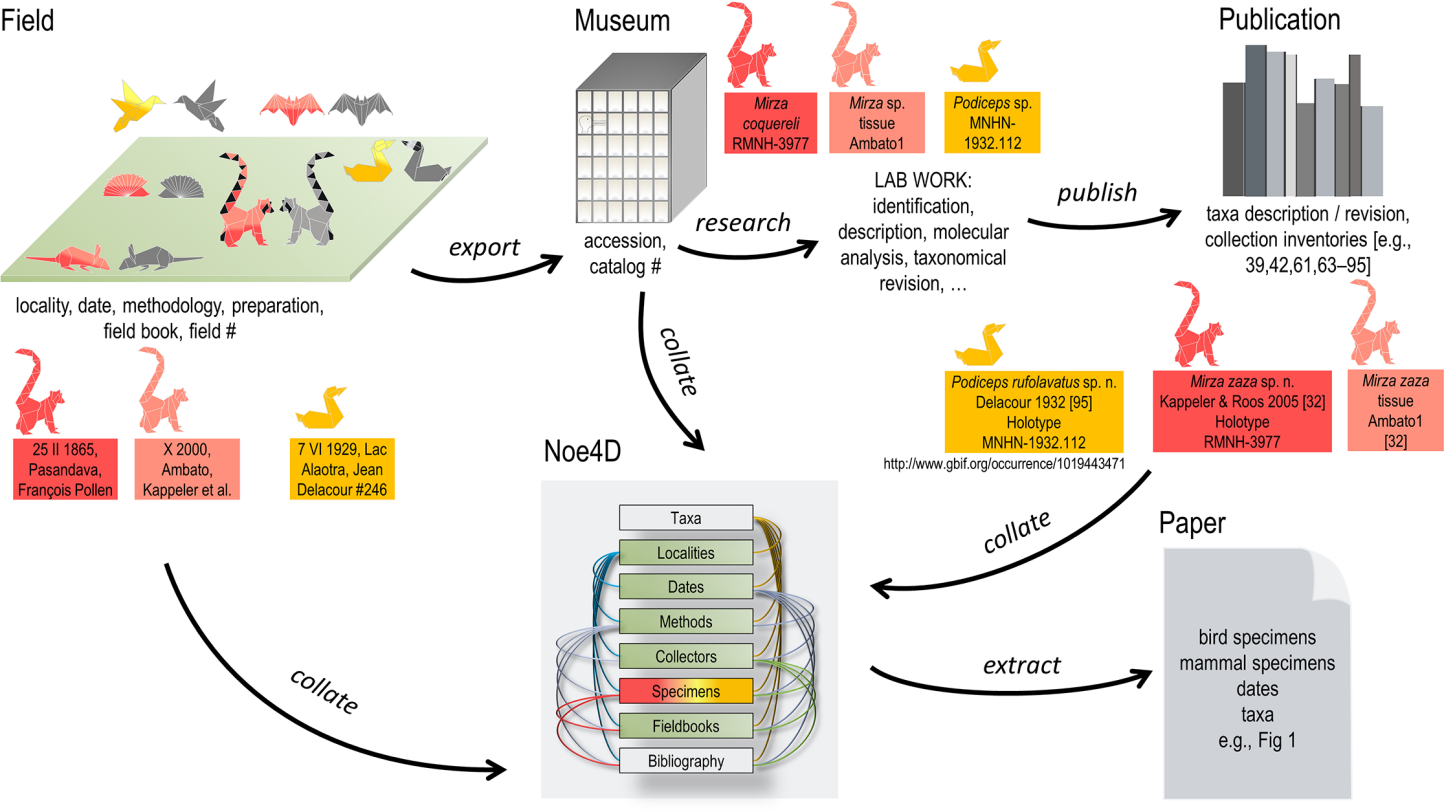
Data and information processing from field, museum and publication [e.g., 32,39,42,61,63–95]; information is collected and collated in the Noe4D database. The red boxes represent an actual case study from a specimen killed in the field on 25 February 1865, and an ear clip saved as a specimen in October 2000, both housed in the Museum of Leiden, NL, and further designated as the holotype of a new species described [32]. The yellow box is a case study from a grebe (*Tachybaptus* sp.) killed during the MZFAA on 7 June 1929, field number 246, accessed in the MNHN and becoming the holotype of a new species described in 1932 [95]. (Grey animals represent wildlife, colored animals represent killed and non-killed specimens with color code as in Fig 1)

This paper builds on searches in the Noe4D database documenting the ecology and taxonomy of the biodiversity of Madagascar’s land vertebrates (birds and mammals) as well as a group of invertebrates (scorpions) [60,61]. The Noe4D database, developed and maintained by the senior author (LW) since 1994, includes data from specimens conserved in natural history museums, museum catalogues, field books associated with the collections, and data extracted from published references (Fig 2). The database is structured in three main modules: (i) systematic, with the specimens linked to the taxa; (ii) bibliography; and (iii) ecology (including locality, time, methodology) [60,62].

We conducted a structured search of published sources in the Noe4D database using the keywords “taxa”, “specimen”, “birds”, “mammals”, “scorpions” and a Boolean combination of these with the term “Madagascar” and “endemic”, to extract taxonomic information of Madagascar endemic species and subspecies, as well as specimens associated with these taxa. The Noe4D database currently contains 50,287 records; a record being a taxon in a given locality at a given time, recorded according to a methodology (e.g., life traps, mist-nets, extractive traps, direct observations). Multiple specimens could therefore represent a single record, and almost half of the records in the database are not based on specimens but on published literature. In the Noe4D database, 22,740 records are based on museum material and 27,547 on publications; out of the 40,979 specimens entered in the database, 27,030 refer to endemic birds and mammals and have been turned into records because they included reliable information on at least the locality and the date.

For verification purposes, we have searched GBIF for Madagascar taxa belonging to the Class Mammalia and Class Aves, and the Order Scorpiones. We found some 33,864 specimens, including 16,693 mammal, 16,410 bird and 761 scorpion specimens. Amongst them, we have only considered specimens identified at least at the species level, belonging to endemic taxa, and bearing information on a collection locality and a date or period. The sample was therefore reduced to 17,115 specimens, including 6,640 mammals, 10,056 birds and 419 scorpions.

## Supporting information

S1 Case The documentation of scorpions requires limited specimen sampling. (PDF)

S1 Fig Description of endemic species and subspecies of birds, mammals, and scorpions over time, and number of specimens of endemic birds and mammals. Specimens and taxa are based on Noe4D (upper portion of graphs) and GBIF (lower portion). Overall, the grand majority of specimens consists of killed specimens, with the non-killed specimens gaining momentum in the last two decades. Since the 1930s only a few new bird taxa have been described, while the description of mammals has surged since the mid-1990s with the description of new species of small mammals in the orders Afrosoricida and Rodentia, and the application of the Phylogenetic Species Concept and new molecular tools to the systematics of lemurs. Since the 1990s, thousands of birds and mammals have been removed from the wild to document the diversity of these two groups in Madagascar. Despite increasing efforts to document the other endemic vertebrates since the 1990s the only class for which we have a good taxonomic knowledge in Madagascar, and have done for over 50 years, is the birds. In total, there have been 92 endemic taxa (species and subspecies) of scorpions, 176 endemic taxa of birds and 219 endemic taxa of mammals described by the end of 2016.
(PDF)

S1 Table Museums of Natural History holding the specimens considered during the present study. (PDF)

S2 Table Endemic species and subspecies of scorpions described since the 1990s. (PDF)

S3 Table The taxa in the new Bernieridae endemic family and the number of birds killed which are documented in the Noe4D database. (The MZFAA took place from April 1929 to May 1931).
(PDF)

## References

[1] Wakeham-Dawson A, Morris S, Tubbs PK, Dalebout ML, Baker CS. Nomenclatural Notes-Type specimens: Dead or alive? Bulletin of zoological Nomenclature. 2002;59(4):282–285.

[2] Wilson DE, Reeder DM, editors. Mammal species of the world: a taxonomic and geographic reference. JHU Press; 2005.

[3] Helgen KM. The mammal family tree. Science. 2011 10 28;334(6055):458–459. doi: 10.1126/science.1214544 22034419 10.1126/science.1214544

[4] Prum RO, Berv JS, Dornburg A, Field DJ, Townsend JP, Lemmon EM, Lemmon AR. A comprehensive phylogeny of birds (Aves) using targeted next-generation DNA sequencing. Nature. 2015 10 7;526:569–573. doi: 10.1038/nature15697 26444237 10.1038/nature15697

[5] Rosauer DF, Jetz W. Phylogenetic endemism in terrestrial mammals. Global Ecology and Biogeography. 2015 2 1;24(2):168–179.

[6] Minteer BA, Collins JP, Love KE, Puschendorf R. Avoiding (re) extinction. Science. 2014 4 18;344(6181):260–261. doi: 10.1126/science.1250953 24744362 10.1126/science.1250953

[7] Brengtson S-A. Breeding ecology and extinction of the Great Auk (*Pinguinus impennis*): Anecdotal evidence and conjectures. The Auk. 1984;101(1):1–12.

[8] Rodriguez-Estrella R, Moreno MC. Rare, fragile species, small populations, and the dilemma of collections. Biodiversity and Conservation. 2006 5 1;15(5):1621–1625.

[9] Krell FT, Wheeler QD. Specimen collection: plan for the future. Science. 2014 5 23;344(6186):815–816. doi: 10.1126/science.344.6186.815 24855246 10.1126/science.344.6186.815

[10] Moratelli R. Wildlife biologists are on the right track: a mammalogist's view of specimen collection. Zoologia (Curitiba). 2014 10;31(5):413–417.

[11] Rocha LA, Aleixo A, Allen G, Almeda F, Baldwin CC, Barclay MV, Bates JM, Bauer AM, Benzoni F, Berns CM, Berumen ML. Specimen collection: An essential tool. Science. 2014 5 23;344(6186):814–815. doi: 10.1126/science.344.6186.814 24855245 10.1126/science.344.6186.814

[12] Haberman KL. On the significance of small dead things. Journal of Natural History Education and Experience. 2015;9:8–12.

[13] Ceríaco LM, Gutiérrez EE, Dubois A. Photography-based taxonomy is inadequate, unnecessary, and potentially harmful for biological sciences. Zootaxa. 2016 11 23;4196(3):435–445. 10.11646/zootaxa.4196.3.9 27988669

[14] Cianferoni F, Bartolozzi L. Warning: potential problems for taxonomy on the horizon? Zootaxa. 2016 7 19;4139(1):128–130. doi: 10.11646/zootaxa.4139.1.8 27470790 10.11646/zootaxa.4139.1.8

[15] Löbl I, Cibois A, Landry B. Describing new species in the absence of sampled specimens: a taxonomist's own-goal. The Bulletin of Zoological Nomenclature. 2016 3;73(1):83–86.

[16] Pape T, Allison A, Bickel DJ, Carlton JT, Dikow T, Donegan T, et al Taxonomy: species can be named from photos. Nature. 2016 9 15;537(7620):307. 10.1038/537307b 27629630

[17] Garraffoni AR, Freitas AV. Photos belong in the taxonomic Code. Science. 2017 2 24;355(6327):805. 10.1126/science.aam7686 28232546

[18] Goodman SM (Ed.). A floral and faunal inventory of the eastern slopes of the Réserve Naturelle Intégrale d'Andringitra, Madagascar: with reference to elevational variation. Fieldiana: Zoology, new series. 1996; 85:1–319.

[19] Goodman SM (Ed.). A floral and faunal inventory of the Parc National de Marojejy, Madagascar: with reference to elevational variation. Fieldiana: Zoology, new series. 2000; 97:1–286.

[20] Lourenço WR, Waeber PO, Wilmé L. The geographical pattern of distribution of the genus Tityobuthus Pocock, 1890, a typical Ananterinae element endemic to Madagascar (Scorpiones: Buthidae). Comptes Rendus Biologies. 2016 10 31;339(9):427–436. 27461558 10.1016/j.crvi.2016.06.003

[21] Umetsu F, Naxara L, Pardini R. Evaluating the efficiency of pitfall traps for sampling small mammals in the Neotropics. Journal of Mammalogy. 2006; 87(4):757–765.

[22] Rand AL. The distribution and habits of Madagascar birds. A summary of the field notes of the Mission Zoologique Franco-Anglo-Américaine à Madagascar. Bulletin American Museum of Natural History. 1936;72:143–499.

[23] Wilmé L. Composition and characteristics of bird communities in Madagascar. In: Lourenço, WR, editor. Biogéographie de Madagascar. Paris: Editions de l'ORSTOM. 1996. pp. 349–362.

[24] Tattersall I. Madagascar's lemurs: Cryptic diversity or taxonomic inflation? Evolutionary Anthropology. 2007;16(1):12–23.

[25] Tattersall I. Understanding species-level primate diversity in Madagascar. Madagascar Conservation & Development. 2013;8(1):7–11.

[26] Jenkins RK, Racey PA. Bats as bushmeat in Madagascar. Madagascar Conservation & Development. 2008;3(1):22–30.

[27] Cibois A, Normand D, Gregory SM, Pasquet E. Bernieridae (Aves: Passeriformes): a family-group name for the Malagasy sylvioid radiation. Zootaxa. 2010 7 30;2554(1):65–68.

[28] Jiguet F. Taxonomy of the Kelp Gull *Larus dominicanus* Lichtenstein inferred from biometrics and wing plumage pattern, including two previously undescribed subspecies. Bulletin-British Ornithologists' Club. 2002;122(1):50–70.

[29] Carleton MD, Goodman SM. Systematic studies of Madagascar's endemic rodents (Muroidea: Nesomyinae): a new genus and species from the Central Highlands. Fieldiana Zoology. 1996:231–256.

[30] Wozencraft WC. A new species of striped mongoose from Madagascar. Journal of mammalogy. 1986 8 1:561–571.

[31] IUCN. The IUCN Red List of Threatened Species. Version 2016–3; 2017. <http://www.iucnredlist.org>

[32] Kappeler PM, Rasoloarison RM, Razafimanantsoa L, Walter L, Roos C. Morphology, behaviour and molecular evolution of giant mouse lemurs (Mirza spp.) Gray, 1870, with description of a new species. Primate Report. 2005;71:3–26.

[33] Thalmann U, Geissmann T. Distribution and Geographic Variation in the Western Woolly Lemur (Avahi occidentalis) with Description of a New Species (A. unicolor. International Journal of Primatology. 2000 12 1;21(6):915–941.

[34] Andriantompohavana R, Lei R, Zaonarivelo JR, Engberg SE, Nalanirina G, McGuire SM, Shore GD, Andrianasolo J, Herrington K, Brenneman RA, Louis EE Jr. Molecular phylogeny and taxonomic revision of the woolly lemurs, genus Avahi (Primates: Lemuriformes). Special Publications of the Museum of Texas Tech University. 2007;51:1–59.

[35] Lei R, Engberg SE, Andriantompohavana R, McGuire SM, Mittermeier RA, Zaonarivelo JR, Brenneman RA, Louis EE Jr. Nocturnal lemur diversity at Masoala National Park. Special Publications of the Museum of Texas Tech University. 2008;53:1–41.

[36] Lei R, McLain AT, Frasier CL, Taylor JM, Bailey CA, Engberg SE, Ginter AL, Nash SD, Randriamampionona R, Groves CP, Mittermeier RA. A new species in the genus Cheirogaleus (Cheirogaleidae). Primate Conservation. 2015 12 15;29:43–54.

[37] Thiele D, Razafimahatratra E, Hapke A. Discrepant partitioning of genetic diversity in mouse lemurs and dwarf lemurs–biological reality or taxonomic bias?. Molecular Phylogenetics and Evolution. 2013;69(3):593–609. doi: 10.1016/j.ympev.2013.07.019 23899779 10.1016/j.ympev.2013.07.019

[38] Frasier CL, Lei R, McLain AT, Taylor JM, Bailey CA, Ginter AL, Nash SD, Randriamampionona R, Groves CP, Mittermeier RA, Louis EE Jr. A new species of dwarf lemur (Cheirogaleidae: Cheirogaleus medius group) from the Ankarana and Andrafiamena–Andavakoera Massifs, Madagascar. Primate Conservation. 2016;30:59–72.

[39] Louis EE Jr, Engberg SE, Lei R, Geng H, Sommer JA, Randriamampionona R, Randriamanana JC, Zaonarivelo JR, Andriantompohavana R, Randria G, Prosper Ramaromilanto B, Rakotoarisoa G, Rooney A, Brenneman RA. Molecular and morphological analyses of the sportive lemurs (Family Megaladapidae: Genus Lepilemur) reveals 11 previously unrecognized species. Special Publications, Museum of Texas Tech University. 2006;49:1–47.

[40] Hotaling S, Foley ME, Lawrence NM, Bocanegra J, Blanco MB, Rasoloarison R, Kappeler PM, Barrett MA, Yoder AD, Weisrock DW. Species discovery and validation in a cryptic radiation of endangered primates: coalescent‐based species delimitation in Madagascar's mouse lemurs. Molecular ecology. 2016 3 1;25(9):2029–2045. doi: 10.1111/mec.13604 26946180 10.1111/mec.13604

[41] Radespiel U, Olivieri G, Rasolofoson DW, Rakotondratsimba G, Rakotonirainy O, Rasoloharijaona S, Randrianambinina B, Ratsimbazafy JH, Ratelolahy F, Randriamboavonjy T, Rasolofoharivelo T, Craul M, Rakotozafy L, Randrianarison RM. Exceptional diversity of mouse lemurs (Microcebus spp.) in the Makira region with the description of one new species. American Journal of Primatology. 2008;70(11):1033–1046. doi: 10.1002/ajp.20592 18626970 10.1002/ajp.20592

[42] Jenkins PD, Carleton MD. Charles Immanuel Forsyth Major's expedition to Madagascar, 1894 to 1896: beginnings of modern systematic study of the island's mammalian fauna. Journal of Natural History. 2005; 39(20):1779–1818.

[43] Mercier J-R. Madagascar moving towards sustainable development. The preparation of the National Environmental Action Plan (NEAP): Was it a false start?. Madagascar Conservation & Development. 2006; 1(1):50–54.

[44] Waeber PO, Wilmé L, Mercier JR, Camara C, Lowry II PP. How Effective Have Thirty Years of Internationally Driven Conservation and Development Efforts Been in Madagascar?. PloS one. 2016 8 17;11(8):e0161115 doi: 10.1371/journal.pone.0161115 27532499 10.1371/journal.pone.0161115 PMC4988661

[45] Lesson RP. Description d'un nouveau genre d'oiseau, l'Eurycère, *Euryceros*. Annales des Sciences Naturelles. 1831;22:421–423.

[46] Gutiérrez EE, Pine RH. Specimen collection crucial to taxonomy. Science. 2017 3 24;355(6331):1275 doi: 10.1126/science.aan0926 28336633 10.1126/science.aan0926

[47] Fontaine B, Perrard A, Bouchet P. 21 years of shelf life between discovery and description of new species. Current Biology. 2012;22(22):R943–R944. doi: 10.1016/j.cub.2012.10.029 23174292 10.1016/j.cub.2012.10.029

[48] Waeber PO, Wilmé L, Ramamonjisoa B, Garcia C, Rakotomalala D, Rabemananjara ZH, Kull CA, Ganzhorn JU, Sorg JP. Dry forests in Madagascar: neglected and under pressure. International Forestry Review. 2015 9 11;17(S2):127–148.

[49] Collen B, McRae L, Loh J, Deinet S, De Palma A, Manley R, Baillie JE. Tracking change in abundance: the living planet index. Biodiversity Monitoring and Conservation: Bridging the Gap between Global Commitment and Local Action. 2013:71–94.

[50] World Wide Fund for Nature. Living Planet Report 2016. Risk and resilience in a new era. WWF International, Gland, Switzerland. 2016. Available at <http://awsassets.panda.org/downloads/lpr_living_planet_report_2016.pdf>

[51] Butchart SH, Walpole M, Collen B, Van Strien A, Scharlemann JP, Almond RE, et al Global biodiversity: indicators of recent declines. Science. 2010;328(5982):1164–1168. doi: 10.1126/science.1187512 20430971 10.1126/science.1187512

[52] Ito R, Rakotondraparany F, Sato H. Non-flying mammalian fauna of Ampijoroa, Ankarafantsika National Park. Madagascar Conservation & Development. 2013;8,1:45–48.

[53] Seregin AP. Making the Russian Flora Visible: Fast Digitisation of the Moscow University Herbarium (MW) in 2015. Taxon. 2016 3 8;65(1):205–207.

[54] Blagoderov V, Kitching IJ, Livermore L, Simonsen TJ, Smith VS. No specimen left behind: industrial scale digitization of natural history collections. ZooKeys. 2012;209:133–146. 10.3897/zookeys.209.3178 PMC3406472 22859884

[55] Hebert PD, Zakharov EV, Prosser SW, Sones JE, McKeown JT, Mantle B, La Salle J. A DNA ‘Barcode Blitz’: Rapid digitization and sequencing of a natural history collection. PLoS One. 2013;10,8(7):e68535 doi: 10.1371/journal.pone.0068535 23874660 10.1371/journal.pone.0068535 PMC3707885

[56] Filardi CE. Why I collected a moustached kingfisher. Audubon) http://www.audubon.org/news/why-i-collected-moustached-kingfisher. 2015.

[57] Lourenço WR, Cloudsley-Thompson JL. Notes on the postembryonic development of *Heteroscorpion opisthacanthoides* (Kraepelin, 1896) (Scorpiones, Heteroscorpionidae) from the Island of Nosy Be in the North of Madagascar. Entomologische Mitteilungen aus dem Zoologischen Museum Hamburg. 2003;14(168):129–136.

[58] Lourenço WR, Leguin E-A, Cloudsley-Thompson JL. The life history of the Malagasy scorpion *Opisthacanthus madagascariensis* Kraepelin, 1894 (Liochelidae). Entomologische Mitteilungen aus dem Zoologischen Museum Hamburg. 2010;15(183):173–182.

[59] Berner PO, Wilmé L. Etude d'impact environnemental: Le rôle des scientifiques et des institutions scientifiques. Akon'ny Ala. 1997;20:2–7.

[60] Wilmé L, Ravokatra M, Dolch R, Schuurman D, Mathieu E, Schuetz H, Waeber PO. Toponyms for centers of endemism in Madagascar. Madagascar Conservation & Development. 2012;7(1):30–40.

[61] Lourenço WR. Scorpions (Chelicerata, Scorpiones). Faune de Madagascar N. 87. Muséum national d’Histoire naturelle, Paris. 1996;106pp.

[62] Hertu O, Elouard J-M. Logiciels NOE et CartoNOE. In: Biodiversité et biotypologie des eaux continentales de Madagascar. Institut de Recherche pour le Développement (IRD) & Centre National de la Recherche pour l'Environnement (CNRE). 2001;361–381.

[63] Bartlett E. List of the mammals and birds collected by Mr. Waters in Madagascar. Proceedings of the Zoological Society of London. 1875;62–69.

[64] Benson CW. The Cambridge collection from the Malagasy region. Bulletin of the British Ornithologists' Club. 1970;90:168–172.

[65] Benson CW. The Cambridge collection from the Malagasy region, Part II. Bulletin of the British Ornithologists' Club. 1971;91:1–7.

[66] Benson CW. Type specimens of bird skins in the University Museum of Zoology, Cambridge, United Kingdom. British Ornithologists' Club Occasional Publication. 1999; No. 4, Cambridge.

[67] Buettner-Janusch J, Tattersall I. An annotated catalogue of Malagasy Primates (Families Lemuridae, Indriidae, Daubentoniidae, Megaladapidae, Cheirogaleidae) in the collections of the American Museum of Natural History. American Museum Novitates. 1985;2834:1–45.

[68] Carleton MD. Systematic studies of Madagascar's endemic rodents (Muroidea: Nesomyinae): Revision of the genus Eliurus. American Museum Novitates. 1994;3087:1–55.

[69] Carleton MD, Schmidt DF. Systematic studies of Madagascar's endemic rodents (Muroidea: Nesomyinae): An annotated gazetteer of collecting localities of known forms. American Museum Novitates. 1990;2987:1–36.

[70] Carleton MD, Smeenk C, Angermann R, Goodman SM. Taxonomy of nesomyine rodents (Muroidea: Nesomyidae: Nesomyinae): Designation of lectotypes and restriction of type localities for species-group taxa in the genus Nesomys Peters. Proceedings of the Biological Society of Washington. 2014;126(4):414–455.

[71] Dekker RWRJ. (Ed.) Type specimens of birds of the National Museum of Natural History, Leiden. Part. 2. Passerines: Eurylaimidae-Eopsaltriidae (Peters's sequence). Nationaal Natuurhistorisch Museum Naturalis Technical Bulletin. 2003; 6:1–142.

[72] Goodman SM, Raheriarisena M, Jansa SA. A new species of Eliurus Milne Edwards, 1885 (Rodentia: Nesomyinae) from the Réserve Spéciale d’Ankarana, northern Madagascar. Bonner Zoologische Beiträge. 2009;56 (3):133–149.

[73] Gray GR. Hand-list of genera and species of birds, distinguishing those contained in the British Museum. Part I. Accipitres, Fissirostres, Tenuirostres and Dentirostres. Trustees of the British Museum (Natural History), London. 1869; i–xx,1–404.

[74] Gray GR. Hand-list of genera and species of birds, distinguishing those contained in the British Museum. Part II. Conirostris, Scansores, Columbæ, and Gallinæ. Trustees of the British Museum (Natural History), London. 1870;i–xv,1–279.

[75] Gray GR. Hand-list of genera and species of birds, distinguishing those contained in the British Museum. Part III. Struthiones, Grallæ, and Anseres, with indices of generic and specific names. Trustees of the British Museum (Natural History), London. 1871; i–xii,1–350.

[76] Hellmayr CE. The ornithological collection of the Zoological Museum in Munich. The Auk. 1928;45(3):293–301.

[77] Jenkins PD. Catalogue of primates in the British Museum (Natural History) and elsewhere in the British Isles. Part IV: Suborder Strepsirrhini, including the subfossil Madagascan lemurs and Family Tarsiidae. British Museum (Natural History), London. 1987;189 pp.

[78] LeCroy M. Type specimens of birds in the American Museum of Natural History. Part 5. Passeriformes: Alaudidae, Hirundinidae, Motacillidae, Campephagidae, Pycnonotidae, Irenidae, Laniidae, Vangidae, Bombycillidae, Dulidae, Cinclidae, Troglodytidae, and Mimidae. Bulletin of the American Museum of Natural History. 2003;278:1–156.

[79] LeCroy M. Type specimens of birds in the American Museum of Natural History. Part 6. Passeriformes: Prunellidae, Turdidae, Orthonychidae, Timaliidae, Paradoxornithidae, Picathartidae, and Polioptilidae. Bulletin of the American Museum of Natural History. 2005;292(1):1–127.

[80] Lönnberg E. The ornithological collection of the Natural History Museum in Stockholm. The Auk. 1926;43(4):434–446.

[81] Ménégaux MA. Liste des oiseaux rapportés en 1906 par M. Geay du sud-ouest de Madagascar. Bulletin du Muséum national d'Histoire naturelle, Paris. 1907;13:104–113.

[82] Milon P. Etude d'une petite collection d'oiseaux du Tsaratanana. Le Naturaliste Malgache. 1951;3(2):167–183.

[83] Pucheran J. Observations sur les types peu connus du Musée de Paris. Rapaces nocturnes. Revue et Magasin de Zoologie Pure et Appliquée. 1849; 2,1:17–29 (footnote).

[84] Rasoloarison RM, Goodman SM, Ganzhorn JU. Taxonomic revision of mouse lemurs (*Microcebus*) in the western portions of Madagascar. International Journal of Primatology. 2000;21(6):963–1019.

[85] Richmond CW. Catalogue of a collection of birds made by Doctor W. L. Abbott in Madagascar, with descriptions of three new species. Proceedings of the United States National Museum. 1897;19:677–694.

[86] Roselaar CS. An inventory of major European bird collections. Bulletin of the British Ornithologists' Club. 2003;123A:253–337.

[87] van den Hoek Ostende LW, Dekker RWRJ, Keijl GO. Type specimens of birds of the National Museum of Natural History, Leiden. Part. 1. Non-Passerines. Nationaal Natuurhistorisch Museum Naturalis Technical Bulletin. 1997;1:1–248.

[88] Voisin C. Liste des spécimens types d'ibis (Threskiornithinés) de la collection du Musée National d'Histoire Naturelle de Paris. Oiseau et Revue Française d'Ornithologie. 1993;63(1):45–53.

[89] Voisin C, Voisin J-F. Un spécimen méconnu de Coua de Dalalande *Coua delalandei* (Temminck). Oiseau et Revue Française d'Ornithologie. 1991;61(4):341–342.

[90] Voisin C, Voisin J-F. Liste des types d’oiseaux des collections du Muséum national d’Histoire naturelle de Paris. 8: Rapaces diurnes (Accipitridés), première partie. Zoosystema. 2001;23(1):173–190.

[91] Voisin C, Voisin J-F. Liste des types d’oiseaux des collections du Muséum national d’Histoire naturelle de Paris. 16: Perroquets (Psittacidae). Zoosystema. 2008;30(2):463–499.

[92] Voisin C, Voisin J-F. List of type specimens of birds in the collections of the Muséum national d’Histoire naturelle (Paris, France). 18. Coraciiformes. Journal of the National Museum (Prague), Natural History Series. 2008;177(1):1–25.

[93] Voisin C, Voisin J-F, Tranier M. Note on Grandidier's lemur collection from Madagascar. Mammalia. 1999;63:535–536.

[94] Wilmé L. Status, distribution and conservation of two Madagascar bird species endemic to Lake Alaotra: Delacour's Grebe *Tachybaptus rufolavatus* and Madagascar Pochard *Aythya innotata*. Biological Conservation. 1994;69(1):15–21.

[95] Delacour J. Les oiseaux de la mission zoologique Franco-Anglo-Américaine à Madagascar. Oiseau et Revue Française d'Ornithologie. 1932;2:1–96.

