# Supplementary material for: On specimen killing in the era of conservation crisis – A quantitative case for modernizing taxonomy and biodiversity inventories
Source: PLoS One. 2017 Sep 13;12(9):e0183903. doi: 10.1371/journal.pone.0183903 (PMC5597100; doi:10.1371/journal.pone.0183903)
Supplement: S1 Case — (PDF) [file pone.0183903.s001.pdf]

S1 Case. The documentation of scorpions requires limited specimen sampling.

The description, and sometimes the identification, of scorpion taxa rely on Museum specimens. This typically applies to most if not all invertebrates (but see [1]), but if hundreds of ants can be collected without affecting a population, narrow ranged scorpion taxa may be threatened by such large scale specimen sampling. We estimate that some 3000 scorpions have been killed in Madagascar since the 1960s.

Scorpions' population dynamics and life history strategies exhibit characteristics of 'equilibrium species', i.e., relatively large adult size, small numbers of better endowed offspring with higher probability of survival [2–6], although a few buthids may exhibit life histories of opportunistic species (smaller size, larger numbers of offspring). Consequently, scorpions generally appear to be unlike most terrestrial invertebrates (most of which have opportunistic life histories) and should be considered among possible exceptions to Pianka's rule [2, also see 3–6]. As equilibrium species, scorpions often exhibit stable and predictable biogeographic patterns with characteristics such as strict habitat and microhabitat dependence. These species characteristically produce single clutches after each insemination, and their intrinsic rates of population growth and mobility are comparatively low. These characteristics favour endemic distributions, and several Malagasy species for example are endemic to single sites. Thus, specificity of habitat and microhabitat conditions and the need for relatively stable and predictable environments make scorpions good indicators of highly endemic regions and relatively undisturbed environments [2–7]. Some of the Malagasy scorpions may be considered threatened, both by the destruction of their environment, but also by intensive collections. These can result from academic activities, but in particular also from non-scientific 'amateurs' who collect both for the 'pet' trade, but also for para-scientific studies. Among these species some in particular can be highlighted, including some large species of the genus *Opisthacanthus*, and especially elements of the endemic family Heteroscorpionidae, genus *Heteroscorpion*. One good example is *H. magnus*, a species endemic to the region of Daraina. These scorpions present extremely long biological cycles and the recovery of their populations following intensive collection may require several decades [8,9].

1. Garraffoni AR, Freitas AV. Photos belong in the taxonomic Code. Science. 2017 Feb 24;355(6327):805.
2. Pianka ER. On r-and K-selection. The American Naturalist. 1970 Nov 1;104(940):592-7.

3. Polis G, Farley R. Characteristics and environmental determinants of natality, growth and maturity in a natural population of the desert scorpion, Paruroctonus mesaensis (Scorpionida: Vaejovidae). Journal of Zoology. 1979 Apr 1;187(4):517–542.

4. Polis GA, Farley RD. Population biology of a desert scorpion: survivorship, microhabitat, and the evolution of life history strategy. Ecology. 1980 Jun 1;61(3):620–629.

5. Polis GA. Ecology. In: Polis GA, editor. The biology of scorpions. Stanford: Stanford University Press; 1990. pp. 247–293.

6. Lourenço WR. Biogéographie évolutive, écologies et les stratégies biodémographiques chez les scorpions néotropicaux. Compte rendu des séances de la société de biogéographie. 1991;67(4):171–190.

7. Noss RF. Indicators for monitoring biodiversity: a hierarchical approach. Conservation biology. 1990 Dec 1;4(4):355–364.

8. Lourenço WR, Cloudsley-Thompson JL. Notes on the postembryonic development of *Heteroscorpion opisthacanthoides* (Kraepelin, 1896) (Scorpiones, Heteroscorpionidae) from the Island of Nosy Be in the North of Madagascar. Entomologische Mitteilungen aus dem Zoologischen Museum Hamburg. 2003;14(168):129–136.

9. Lourenço WR, Leguin E-A, Cloudsley-Thompson JL. The life history of the Malagasy scorpion *Opisthacanthus madagascariensis* Kraepelin, 1894 (Liochelidae). Entomologische Mitteilungen aus dem Zoologischen Museum Hamburg. 2010;15(183):173–182.
