# Supplementary material for: On specimen killing in the era of conservation crisis – A quantitative case for modernizing taxonomy and biodiversity inventories
Source: PLoS One. 2017 Sep 13;12(9):e0183903. doi: 10.1371/journal.pone.0183903 (PMC5597100; doi:10.1371/journal.pone.0183903)
Supplement: S1 Fig — Specimens and taxa are based on Noe4D (upper portion of graphs) and GBIF (lower portion). Overall, the grand majority of specimens consists of killed specimens, with the non-killed specimens gaining momentum in the last two decades. Since the 1930s only a few new bird taxa have been described, while the description of mammals has surged since the mid-1990s with the description of new species of small mammals in the orders Afrosoricida and Rodentia, and the application of the Phylogenetic Species Concept and new molecular tools to the systematics of lemurs. Since the 1990s, thousands of birds and mammals have been removed from the wild to document the diversity of these two groups in Madagascar. Despite increasing efforts to document the other endemic vertebrates since the 1990s the only class for which we have a good taxonomic knowledge in Madagascar, and have done for over 50 years, is the birds. In total, there have been 92 endemic taxa (species and subspecies) of scorpions, 176 endemic taxa of birds and 219 endemic taxa of mammals described by the end of 2016. (PDF) [file pone.0183903.s002.pdf]

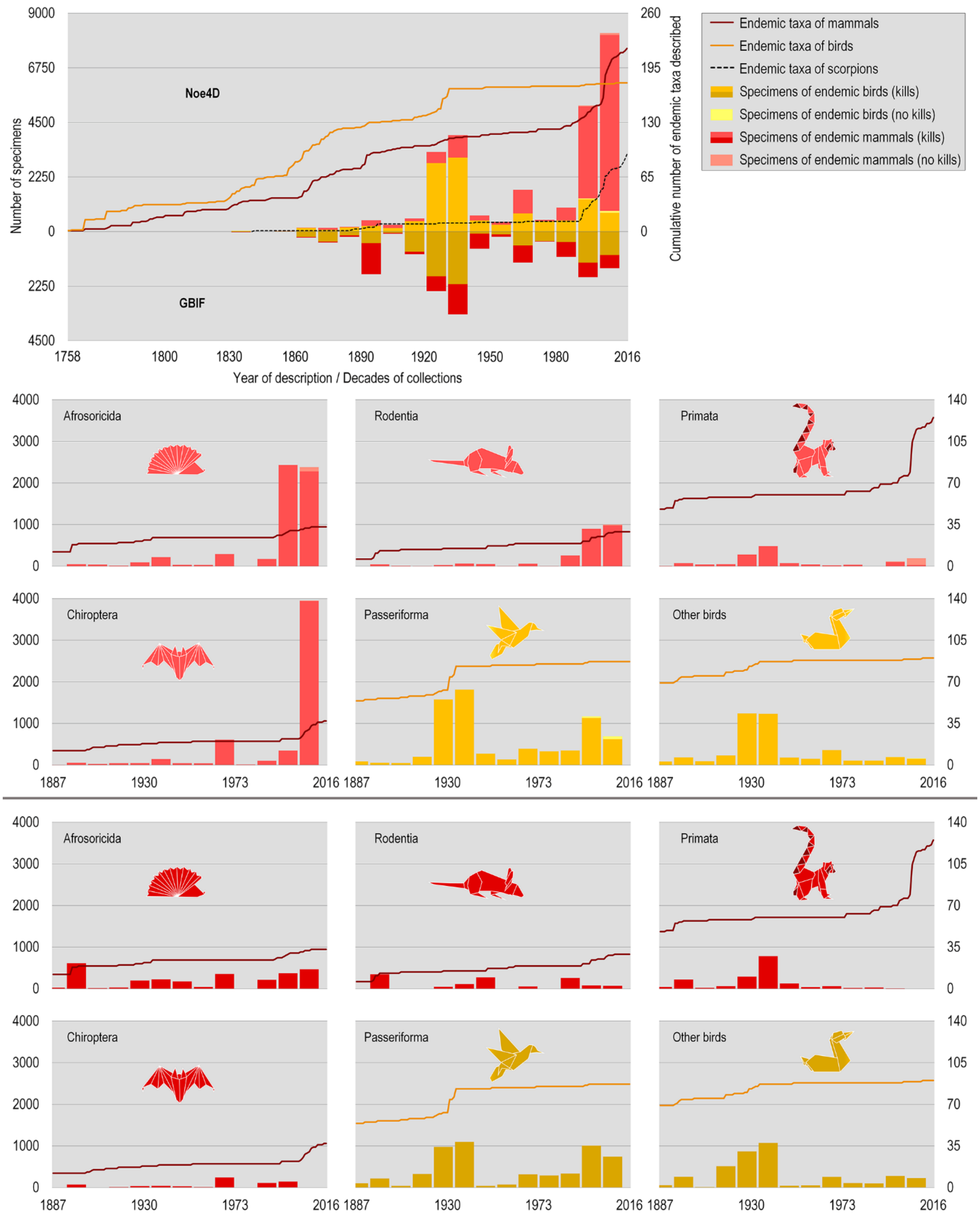

S1 Fig. Description of endemic species and subspecies of birds, mammals, and scorpions over time, and number of specimens of endemic birds and mammals. Specimens and taxa are based on Noe4D (upper portion of graphs) and GBIF (lower portion). Overall, the grand majority of specimens consists of killed specimens, with the non-killed specimens gaining momentum in the last two decades. Since the 1930s only a few new bird taxa have been described, while the description of mammals has surged since the mid-1990s with the description of new species of small mammals in the orders Afrosoricida and Rodentia, and the application of the Phylogenetic Species Concept and new molecular tools to the systematics of lemurs. Since the 1990s, thousands of birds and mammals have been removed from the wild to document the diversity of these two groups in Madagascar. Despite increasing efforts to document the other endemic vertebrates since the 1990s the only class for which we have a good taxonomic knowledge in Madagascar, and have done for over 50 years, is the birds. In total, there have been 92 endemic taxa (species and subspecies) of scorpions, 176 endemic taxa of birds and 219 endemic taxa of mammals described by the end of 2016.

Values used to build Fig1 and S1 Fig

	Endemic species and subspecies described							
Year	Scorpionida	Aves	Mammalia	Afrosoricida	Primata	Chiroptera	Carnivora	Rodentia
1758		1	1	0	1	0	0	0
1759		0	0	0	0	0	0	0
1760		1	0	0	0	0	0	0
1761		0	0	0	0	0	0	0
1762		0	0	0	0	0	0	0
1763		0	0	0	0	0	0	0
1764		0	0	0	0	0	0	0
1765		0	0	0	0	0	0	0
1766		12	2	0	3	0	0	0
1767		0	0	0	0	0	0	0
1768		0	0	0	0	0	0	0
1769		0	0	0	0	0	0	0
1770		0	0	0	0	0	0	0
1771		1	0	0	0	0	0	0
1772		0	0	0	0	0	0	0
1773		0	0	0	0	0	0	0
1774		0	0	0	0	0	0	0
1775		0	0	0	0	0	0	0
1776		9	1	0	0	0	1	0
1777		0	1	0	1	0	0	0
1778		0	2	2	0	0	0	0
1779		0	0	0	0	0	0	0
1780		0	0	0	0	0	0	0
1781		0	0	0	0	0	0	0
1782		0	0	0	0	0	0	0
1783		1	0	0	0	0	0	0
1784		0	0	0	0	0	0	0
1785		0	0	0	0	0	0	0
1786		3	0	0	0	0	0	0
1787		0	0	0	0	0	0	0
1788		1	4	0	4	0	2	0
1789		2	0	0	0	0	0	0
1790		0	0	0	0	0	0	0
1791		0	0	0	0	0	0	0
1792		0	1	0	2	0	0	0
1793		1	0	0	0	0	0	0
1794		0	0	0	0	0	0	0
1795		0	1	0	2	0	0	0
1796		0	2	0	3	0	0	0
1797		0	0	0	0	0	0	0
1798		0	1	1	0	0	0	0
1799		0	1	0	1	0	0	0
1800		0	0	0	0	0	0	0
1801		0	0	0	0	0	0	0
1802		0	0	0	0	0	0	0
1803		0	2	0	0	2	0	0
1804		0	0	0	0	0	0	0
1805		0	0	0	0	0	0	0
1806		0	0	0	0	0	0	0

	Endemic species and subspecies described							
Year	Scorpionida	Aves	Mammalia	Afrosoricida	Primata	Chiroptera	Carnivora	Rodentia
1807		0	0	0	0	0	0	0
1808		0	0	0	0	0	0	0
1809		0	0	0	0	0	0	0
1810		0	0	0	0	0	0	0
1811		1	0	0	0	0	0	0
1812		0	4	0	4	0	0	0
1813		1	1	0	0	1	0	0
1814		0	0	0	0	0	0	0
1815		0	0	0	0	0	0	0
1816		0	0	0	0	0	0	0
1817		0	0	0	0	0	0	0
1818		0	1	0	0	1	0	0
1819		0	0	0	0	0	0	0
1820		0	1	0	0	0	1	0
1821		0	0	0	0	0	0	0
1822		0	0	0	0	0	0	0
1823		1	0	0	0	0	0	0
1824		0	0	0	0	0	0	0
1825		0	0	0	0	0	0	0
1826		0	0	0	0	0	0	0
1827		1	0	0	0	0	0	0
1828		0	0	0	0	0	0	0
1829		0	0	0	0	0	0	0
1830		0	0	0	0	0	0	0
1831		1	0	0	0	0	0	0
1832		2	1	0	2	0	0	0
1833		2	2	0	1	0	1	0
1834		4	2	0	1	1	0	0
1835		0	1	0	0	0	2	0
1836		3	0	0	0	0	0	0
1837		0	2	0	0	0	3	0
1838		3	1	1	0	0	0	0
1839		1	1	0	2	0	0	0
1840		1	0	0	0	0	0	0
1841		0	0	0	0	0	0	0
1842		0	1	0	1	0	0	0
1843		2	0	0	0	0	0	0
1844	1	0	0	0	0	0	0	0
1845	0	7	0	0	0	0	0	0
1846	0	1	0	0	0	0	0	0
1847	0	0	0	0	0	0	0	0
1848	0	0	0	0	0	0	0	0
1849	0	1	0	0	0	0	0	0
1850	0	1	1	0	1	0	0	0
1851	0	0	1	0	2	0	0	0
1852	0	0	1	0	1	0	0	0
1853	0	0	0	0	0	0	0	0
1854	0	1	0	0	0	0	0	0
1855	0	0	0	0	0	0	0	0
1856	0	0	0	0	0	0	0	0
1857	0	0	0	0	0	0	0	0

	Endemic species and subspecies described							
Year	Scorpionida	Aves	Mammalia	Afrosoricida	Primata	Chiroptera	Carnivora	Rodentia
1858	0	1	0	0	0	0	0	0
1859	0	0	0	0	0	0	0	0
1860	0	8	0	0	0	0	0	0
1861	0	2	0	0	0	0	0	0
1862	0	2	0	0	0	0	0	0
1863	0	4	0	0	0	0	0	0
1864	0	0	0	0	0	0	0	0
1865	0	1	1	0	0	1	0	0
1866	0	3	1	0	0	1	0	0
1867	0	8	6	0	7	0	1	0
1868	0	1	0	0	0	0	0	0
1869	0	0	2	0	0	1	0	1
1870	0	3	5	1	2	1	0	1
1871	0	4	5	0	6	0	0	0
1872	0	0	1	2	0	0	0	0
1873	0	1	0	0	0	0	0	0
1874	0	0	1	0	0	1	0	0
1875	0	6	3	1	1	0	0	1
1876	0	0	0	0	0	0	0	0
1877	0	3	0	0	0	0	0	0
1878	0	1	1	0	0	1	0	0
1879	0	4	2	0	0	0	0	2
1880	0	0	0	0	0	0	0	0
1881	0	2	1	0	0	1	0	0
1882	0	1	3	3	0	0	0	0
1883	0	1	0	0	0	0	0	0
1884	0	0	1	1	0	0	0	0
1885	0	1	1	0	0	0	0	1
1886	0	0	0	0	0	0	0	0
1887	0	0	0	0	0	0	0	0
1888	0	0	0	0	0	0	0	0
1889	1	0	0	0	0	0	0	0
1890	1	0	1	0	1	0	0	0
1891	0	1	0	0	0	0	0	0
1892	0	0	0	0	0	0	0	0
1893	0	0	0	0	0	0	0	0
1894	1	1	4	0	6	0	0	0
1895	0	1	1	0	0	0	0	1
1896	1	2	11	6	1	0	0	4
1897	0	2	0	0	0	0	0	0
1898	0	0	2	0	1	0	0	2
1899	0	0	1	1	0	0	0	0
1900	1	0	0	0	0	0	0	0
1901	3	0	0	0	0	0	0	0
1902	0	0	0	0	0	0	0	0
1903	0	1	1	0	0	1	0	0
1904	0	0	0	0	0	0	0	0
1905	0	0	1	0	0	1	0	0
1906	0	0	1	0	0	1	0	0
1907	0	0	0	0	0	0	0	0
1908	0	1	1	0	0	0	0	1

	Endemic species and subspecies described							
Year	Scorpionida	Aves	Mammalia	Afrosoricida	Primata	Chiroptera	Carnivora	Rodentia
1909	0	0	0	0	0	0	0	0
1910	0	0	1	0	1	0	0	0
1911	0	0	0	0	0	0	0	0
1912	0	1	1	0	0	1	0	0
1913	0	0	0	0	0	0	0	0
1914	0	0	0	0	0	0	0	0
1915	0	0	0	0	0	0	0	0
1916	0	0	0	0	0	0	0	0
1917	0	0	0	0	0	0	0	0
1918	0	3	2	1	0	1	0	0
1919	0	0	0	0	0	0	0	0
1920	0	1	0	0	0	0	0	0
1921	0	0	0	0	0	0	0	0
1922	0	1	0	0	0	0	0	0
1923	0	0	0	0	0	0	0	0
1924	0	1	0	0	0	0	0	0
1925	0	1	0	0	0	0	0	0
1926	0	1	1	1	0	0	0	0
1927	0	1	0	0	0	0	0	0
1928	0	1	1	0	0	0	0	1
1929	1	3	2	0	0	1	1	0
1930	0	0	1	1	0	0	0	0
1931	0	12	1	0	1	0	0	0
1932	0	1	1	0	1	0	0	0
1933	0	2	0	0	0	0	0	0
1934	0	9	2	2	0	0	0	0
1935	0	0	0	0	0	0	0	0
1936	0	0	0	0	0	0	0	0
1937	0	0	1	0	0	1	0	0
1938	0	0	0	0	0	0	0	0
1939	0	0	0	0	0	0	0	0
1940	0	0	0	0	0	0	0	0
1941	0	0	1	0	0	0	1	0
1942	0	0	0	0	0	0	0	0
1943	0	0	0	0	0	0	0	0
1944	0	0	0	0	0	0	0	0
1945	0	0	0	0	0	0	0	0
1946	1	0	0	0	0	0	0	0
1947	0	0	0	0	0	0	0	0
1948	0	0	0	0	0	0	0	0
1949	0	0	2	0	0	0	0	2
1950	0	1	0	0	0	0	0	0
1951	0	1	0	0	0	0	0	0
1952	0	0	0	0	0	0	0	0
1953	0	0	1	0	0	1	0	0
1954	0	0	0	0	0	0	0	0
1955	0	0	0	0	0	0	0	0
1956	0	0	0	0	0	0	0	0
1957	0	0	0	0	0	0	0	0
1958	0	0	0	0	0	0	0	0
1959	0	0	1	0	0	0	0	1

	Endemic species and subspecies described							
Year	Scorpionida	Aves	Mammalia	Afrosoricida	Primata	Chiroptera	Carnivora	Rodentia
1960	0	0	0	0	0	0	0	0
1961	0	0	0	0	0	0	0	0
1962	0	0	1	0	0	0	0	1
1963	0	0	0	0	0	0	0	0
1964	0	0	0	0	0	0	0	0
1965	0	0	0	0	0	0	0	0
1966	0	0	0	0	0	0	0	0
1967	0	0	0	0	0	0	0	0
1968	0	0	0	0	0	0	0	0
1969	1	0	0	0	0	0	0	0
1970	0	0	0	0	0	0	0	0
1971	0	0	1	0	0	0	1	0
1972	0	1	0	0	0	0	0	0
1973	0	0	0	0	0	0	0	0
1974	0	0	0	0	0	0	0	0
1975	0	0	3	0	3	0	0	0
1976	0	0	0	0	0	0	0	0
1977	0	0	0	0	0	0	0	0
1978	0	0	0	0	0	0	0	0
1979	0	0	0	0	0	0	0	0
1980	0	0	0	0	0	0	0	0
1981	0	0	0	0	0	0	0	0
1982	0	0	0	0	0	0	0	0
1983	0	0	0	0	0	0	0	0
1984	0	0	0	0	0	0	0	0
1985	0	0	0	0	0	0	0	0
1986	0	0	1	0	0	0	1	0
1987	0	0	2	0	2	0	0	0
1988	0	0	1	0	1	0	0	0
1989	0	0	0	0	0	0	0	0
1990	0	0	0	0	0	0	0	0
1991	0	0	3	0	3	0	0	0
1992	0	0	1	1	0	0	0	0
1993	0	0	1	1	0	0	0	0
1994	0	0	2	0	0	0	0	2
1995	4	1	2	0	0	2	0	0
1996	10	0	1	1	0	0	0	0
1997	1	1	2	1	1	0	0	0
1998	1	0	4	1	0	0	0	3
1999	4	0	1	1	0	0	0	0
2000	4	0	4	0	4	0	0	0
2001	1	0	2	0	1	0	0	1
2002	2	1	1	0	1	0	0	0
2003	3	0	0	0	0	0	0	0
2004	8	0	2	1	0	1	0	0
2005	1	0	7	0	3	2	0	2
2006	15	0	27	1	25	3	0	1
2007	1	0	7	0	6	1	0	0
2008	5	0	6	0	5	1	0	0
2009	2	0	6	1	1	3	0	1
2010	1	0	2	0	0	1	1	0

	Endemic species and subspecies described							
Year	Scorpionida	Aves	Mammalia	Afrosoricida	Primata	Chiroptera	Carnivora	Rodentia
2011	0	1	1	0	1	0	0	0
2012	1	0	2	0	0	2	0	0
2013	1	0	3	0	3	0	0	0
2014	4	0	0	0	0	0	0	0
2015	5	0	2	0	1	1	0	0
2016	6	0	3	0	4	0	0	0

Specimens

Noe4D		Aves		Mammalia	
Decades	Scorpions	kill	no kill	kill	no kill
1750	0	0	0	0	0
1760	0	0	0	0	0
1770	0	0	0	0	0
1780	0	0	0	0	0
1790	0	0	0	0	0
1800	0	0	0	0	0
1810	0	0	0	0	0
1820	0	5	0	0	0
1830	0	21	0	0	0
1840	0	1	0	0	0
1850	0	6	0	2	0
1860	0	145	0	18	0
1870	0	72	0	80	0
1880	0	174	0	21	0
1890	3	235	0	229	0
1900	1	141	0	134	0
1910	0	430	0	112	0
1920	0	2818	0	462	1
1930	0	3043	0	932	0
1940	1	452	0	212	0
1950	1	281	0	115	0
1960	2	747	0	979	0
1970	2	429	0	53	0
1980	0	452	0	534	0
1990	45	1323	40	3792	19
2000	270	770	81	7250	395
Totals	325	11545	121	14925	415

Noe4D	Afrosoricida		Primata		Chiroptera		Carnivora		Rodentia	
Decades	kill	no kill	kill	no kill	kill	no kill	kill	no kill	kill	no kill
1750	0	0	0	0	0	0	0	0	0	0
1760	0	0	0	0	0	0	0	0	0	0
1770	0	0	0	0	0	0	0	0	0	0
1780	0	0	0	0	0	0	0	0	0	0
1790	0	0	0	0	0	0	0	0	0	0
1800	0	0	0	0	0	0	0	0	0	0
1810	0	0	0	0	0	0	0	0	0	0
1820	0	0	0	0	0	0	0	0	0	0
1830	0	0	0	0	0	0	0	0	0	0
1840	0	0	0	0	0	0	0	0	0	0
1850	0	0	2	0	0	0	0	0	0	0
1860	0	0	12	0	6	0	0	0	0	0
1870	8	0	42	0	7	0	16	0	7	0
1880	3	0	8	0	5	0	2	0	3	0
1890	50	0	74	0	56	0	2	0	47	0
1900	41	0	42	0	24	0	15	0	12	0
1910	13	0	49	0	44	0	1	0	5	0
1920	90	0	277	1	45	0	24	0	26	0
1930	217	0	483	0	141	0	30	0	61	0
1940	31	0	74	0	42	0	14	0	51	0
1950	27	0	43	0	39	0	0	0	6	0
1960	291	0	19	0	609	0	3	0	57	0
1970	0	0	35	0	13	0	1	0	4	0
1980	171	0	2	0	101	0	1	0	259	0
1990	2433	0	96	19	344	0	21	0	898	0
2000	2279	100	25	167	3942	111	25	1	978	21
Totals	5654	100	1283	187	5418	111	155	1	2414	21

Noe4D	Non Passeriforma		Passeriforma	
Decades	kill	no kill	kill	no kill
1750	0	0	0	0
1760	0	0	0	0
1770	0	0	0	0
1780	0	0	0	0
1790	0	0	0	0
1800	0	0	0	0
1810	0	0	0	0
1820	4	0	1	0
1830	16	0	5	0
1840	1	0	0	0
1850	5	0	1	0
1860	58	0	87	0
1870	48	0	24	0
1880	86	0	88	0
1890	180	0	55	0
1900	91	0	50	0
1910	231	0	199	0
1920	1241	0	1577	0
1930	1230	0	1813	0
1940	179	0	273	0
1950	147	0	134	0
1960	357	0	390	0
1970	103	0	326	0
1980	103	0	349	0
1990	191	3	1132	37
2000	149	12	621	69
Totals	4420	15	7125	106

GBIF Decades	Scorpionida	Aves	Mammalia
1750			
1760			
1770			
1780			
1790			
1800			
1810			
1820	0	1	1
1830	0	14	15
1840	0	4	1
1850	0	3	5
1860	0	229	18
1870	0	418	34
1880	3	154	70
1890	2	477	1281
1900	1	52	41
1910	0	834	102
1920	0	1844	616
1930	1	2170	1244
1940	0	83	625
1950	1	121	96
1960	10	570	713
1970	1	395	24
1980	0	433	612
1990	67	1283	601
2000	333	971	541
Totals	419	10056	6640

GBIF Decades	Mammalia Afrosoricida	Primata	Chiroptera	Carnivora	Rodentia
1750					
1760					
1770					
1780					
1790					
1800					
1810					
1820	0	1	0	0	0
1830	5	3	0	7	0
1840	1	0	0	0	0
1850	0	2	0	1	2
1860	1	13	2	2	0
1870	5	24	0	3	2
1880	23	43	0	4	0
1890	613	224	70	30	344
1900	13	20	1	6	1
1910	29	57	10	6	0
1920	195	291	33	51	46
1930	226	780	41	85	112
1940	176	124	27	28	270
1950	42	39	12	2	1
1960	353	57	242	9	52
1970	3	17	0	4	0
1980	213	29	108	3	259
1990	372	7	142	1	79
2000	468	1	1	0	71
Totals	2738	1732	689	242	1239

GBIF Decades	Aves Non Passeriforma	Passeriforma
1750		
1760		
1770		
1780		
1790		
1800		
1810		
1820	0	1
1830	11	3
1840	1	3
1850	1	2
1860	139	90
1870	235	183
1880	55	99
1890	259	218
1900	11	41
1910	511	323
1920	869	975
1930	1072	1098
1940	43	40
1950	52	69
1960	254	316
1970	107	288
1980	97	336
1990	277	1006
2000	228	743
Totals	4222	5834
