# Supplementary material for: On specimen killing in the era of conservation crisis – A quantitative case for modernizing taxonomy and biodiversity inventories
Source: PLoS One. 2017 Sep 13;12(9):e0183903. doi: 10.1371/journal.pone.0183903 (PMC5597100; doi:10.1371/journal.pone.0183903)
Supplement: S1 Table — (PDF) [file pone.0183903.s003.pdf]

S1 Table. Museums of Natural History holding the specimens considered during the present study.

AIMZ	Anthropological Institute and Museum of the University of Zurich, Zürich, Switzerland
AMNH	American Museum of Natural History, New York, U.S.A.
BMNH	The Natural History Museum, London, U.K.
CAS	California Academy of Sciences, San Francisco, CA, U.S.A.
DLC	Duke Lemur Center, Durham, NC, U.S.A.
FMNH	Field Museum of Natural History, Chicago, IL, U.S.A.
GBP	Gene Bank of Primates, Deutsches Primatenzentrum, Göttingen, Germany
IZUVMH	Institute of Zoology of the University of Veterinary Medicine Hannover, Hannover, Germany
JGUMainz	Institut für Anthropologie, Johannes Gutenberg-Universität Mainz, Mainz, Germany
LACM	Los Angeles County Museum, Los Angeles, CA, U.S.A.
LMCM	Liverpool Merseyside County Museums, Liverpool, U.K.
LSUMZ	Louisiana State University Museum of Natural Science, Baton Rouge, Louisiana, U.S.A.
MCZ	Museum of Comparative Zoology, Harvard University, Cambridge, MA, U.S.A.
MHNGenève	Museum d'Histoire Naturelle Genève, Genève, Suisse
MHNGrenoble	Museum d'Histoire Naturelle de Grenoble, Grenoble, France
MHNLaRochelle	Museum d'Histoire Naturelle de La Rochelle, La Rochelle, France
MHNLille	Museum d'Histoire Naturelle de Lille, Lille, France
MHNNantes	Museum d'Histoire Naturelle de Nantes, Nantes, France
MHNToulouse	Museum d'Histoire Naturelle de Toulouse, Toulouse, France
MNHN	Museum National d'Histoire Naturelle, Paris, France
MRSN	Museo Regionale di Scienze Naturali, Torino, Italia
MVZ	Museum of Vertebrate Zoology, Berkeley, CA, U.S.A.
MZSA	Musée Zoologique de l'Université Louis Pasteur et de la Ville de Strasbourg, Strasbourg, France
MZUN	Musée de Zoologie Université de Nancy, Nancy, France
NHMW	Naturhistorisches Museum Wien, Wien, Austria
NMBasel	Naturhistorisches Museum Basel, Basel, Switzerland
PBZT	Parc Botanique et Zoologique de Tzimbazaza, Antananarivo, Madagascar
RMNH	Rijksmuseum van Natuurlijke Historie [currently Nationaal Natuurhistorisch Museum Naturalis -NNM], Leiden, The Netherlands

ROM	Royal Ontario Museum of Zoology, Ontario, Canada
RSM	Royal Scottish Museum, Edinburgh, U.K.
SMF	Forschungsinstitut und Naturmuseum Senckenberg, Frankfurt, Germany
SMNS	Staatliches Museum für Naturkunde in Stuttgart, Stuttgart, Germany
TTU	The Museum of Texas Tech University, Lubbock, TX, U.S.A.
UADBA	Université Antananarivo, Département Biologie Animale, Antananarivo, Madagascar
UM	Université de Mahajanga, Mahajanga, Madagascar
UMMZ	University of Michigan, Museum of Zoology, Ann Arbor, MI, U.S.A.
USNM	National Museum of Natural History, Smithsonian Institution [United States National Museum], Washington, DC, U.S.A.
UWZM	University of Wisconsin Zoological Museum, Madison, WI, U.S.A.
UZMC	Universitet Zoologisk Museum Copenhagen, Copenhagen, Denmark
WFVZ	Western Foundation of Vertebrate Zoology, Los Angeles, CA, U.S.A.
ZFMK	Zoologischen Forschungsinstituts und Museums Alexander Koënig, Bonn, Germany
ZMA	Zoölogisch Museum Amsterdam, Amsterdam, The Netherlands
ZMB	Zoologishes Museum der Universität, Berlin, Berlin, Germany
ZMH	Zoologishes Museum der Universität Hamburg, Hamburg, Germany
