# Supplementary material for: On specimen killing in the era of conservation crisis – A quantitative case for modernizing taxonomy and biodiversity inventories
Source: PLoS One. 2017 Sep 13;12(9):e0183903. doi: 10.1371/journal.pone.0183903 (PMC5597100; doi:10.1371/journal.pone.0183903)
Supplement: S2 Table — (PDF) [file pone.0183903.s004.pdf]

S2 Table. Endemic species and subspecies of scorpions described between 1990 and 2016.

Family	Species or subspecies	Descriptor	Reference
Buthidae	*Grosphus ankarafantsika*	Lourenço 2003	1
Buthidae	*Grosphus ankarana*	Lourenço, Goodman 2003	2
Buthidae	*Grosphus bicolor*	Lourenço 2012	3
Buthidae	*Grosphus darainensis*	Lourenço, Goodman, Ramilijaona 2004	4
Buthidae	*Grosphus eliseanneae*	Lourenço, Wilmé 2016	5
Buthidae	*Grosphus feti*	Lourenço 1996	6
Buthidae	*Grosphus ganzhorni*	Lourenço, Wilmé, Waeber 2016	7
Buthidae	*Grosphus goudoti*	Lourenço, Goodman 2006	8
Buthidae	*Grosphus hirtus garciai*	Lourenço 2001	9
Buthidae	*Grosphus intertidalis*	Lourenço 1999	10
Buthidae	*Grosphus magalieae*	Lourenço 2014	11
Buthidae	*Grosphus mahafaliensis*	Lourenço, Goodman, Ramilijaona 2004	4
Buthidae	*Grosphus makay*	Lourenço, Wilmé 2015	12
Buthidae	*Grosphus mandena*	Lourenço 2005	13
Buthidae	*Grosphus olgae*	Lourenço 2004	14
Buthidae	*Grosphus polskyi*	Lourenço, Qi, Goodman 2007	15
Buthidae	*Grosphus rossii*	Lourenço 2013	16
Buthidae	*Grosphus sabineae*	Lourenço, Wilmé 2016	5
Buthidae	*Grosphus simoni*	Lourenço, Goodman, Ramilijaona 2004	4
Buthidae	*Grosphus tavaratra*	Lourenço, Soarimalala, Goodman 2009	17
Buthidae	*Grosphus voahangyae*	Lourenço, Wilmé 2015	18
Buthidae	*Grosphus waeberi*	Lourenço, Wilmé 2016	5
Buthidae	*Neogrosphus andrafiabe*	Lourenço, Wilmé, Waeber 2015	19
Buthidae	*Neogrosphus blanci*	Lourenço 1996	6
Buthidae	*Pseudouroplectes betschi*	Lourenço 1995	20
Buthidae	*Pseudouroplectes lalyae*	Lourenço, Ythier 2010	21
Buthidae	*Pseudouroplectes maculatus*	Lourenço, Goodman 2006	22
Buthidae	*Pseudouroplectes pidgeoni*	Lourenço, Goodman 1999	23

Family	Species or subspecies	Descriptor	Reference
Buthidae	*Pseudouroplectes tsingy*	Lourenço, Wilmé, Waeber 2015	24
Buthidae	*Tityobuthus antsingy*	Lourenço, Goodman 2004	25
Buthidae	*Tityobuthus betschi*	Lourenço, Qi, Goodman 2008	26
Buthidae	*Tityobuthus chelbergorum*	Lourenço, Qi, Goodman 2008	26
Buthidae	*Tityobuthus darainensis*	Lourenço, Goodman 2002	27
Buthidae	*Tityobuthus dastychi*	Lourenço 1997	28
Buthidae	*Tityobuthus griswoldi*	Lourenço 2000	29
Buthidae	*Tityobuthus guillaumeti*	Lourenço 1995	20
Buthidae	*Tityobuthus ivohibe*	Lourenço, Goodman 1999	30
Buthidae	*Tityobuthus judsoni*	Lourenço 1996	31
Buthidae	*Tityobuthus lokobe*	Lourenço, Wilmé, Waeber 2016	32
Buthidae	*Tityobuthus manonae*	Lourenço 2000	29
Buthidae	*Tityobuthus mccarteri*	Lourenço, Qi, Goodman 2008	26
Buthidae	*Tityobuthus monodi*	Lourenço 2000	29
Buthidae	*Tityobuthus pallidus*	Lourenço 2004	33
Buthidae	*Tityobuthus parrilloi*	Lourenço 1996	6
Buthidae	*Tityobuthus petrae*	Lourenço 1996	6
Buthidae	*Tityobuthus pococki*	Lourenço 1995	20
Buthidae	*Tityobuthus rakotondravonyi*	Lourenço, Goodman 2003	27
Microcharmidae	*Neoprotobuthus intermedius*	Lourenço 2000	34
Microcharmidae	*Microcharmus pauliani*	(Lourenço 2004)	35
Microcharmidae	*Microcharmus p. pauliani*	(Lourenço 2004)	35
Microcharmidae	*Microcharmus pauliani namoroka*	Lourenço, Goodman, Fisher 2006	36
Microcharmidae	*Microcharmus pauliani ambre*	Lourenço, Goodman, Fisher 2006	36
Microcharmidae	*Microcharmus bemaraha*	Lourenço, Goodman, Fisher 2006	36
Microcharmidae	*Microcharmus cloudsleythompsoni*	Lourenço 1995	20
Microcharmidae	*Microcharmus confluenciatus*	Lourenço, Goodman, Fisher 2006	36
Microcharmidae	*Microcharmus duhemi*	Lourenço, Goodman, Fisher 2006	36
Microcharmidae	*Microcharmus fisheri*	Lourenço 1998	37
Microcharmidae	*Microcharmus hauseri*	Lourenço 1996	38

Family	Species or subspecies	Descriptor	Reference
Microcharmidae	*Microcharmus jussarae*	Lourenço 1996	6
Microcharmidae	*Microcharmus maculatus*	Lourenço, Goodman, Fisher 2006	36
Microcharmidae	*Microcharmus madagascariensis*	Lourenço 1999	39
Microcharmidae	*Microcharmus sabineae*	Lourenço 1996	6
Microcharmidae	*Microcharmus variegatus*	Lourenço, Goodman, Fisher 2006	36
Microcharmidae	*Microcharmus violaceous*	Lourenço, Goodman, Fisher 2006	36
Heteroscorpionidae	*Heteroscorpion goodmani*	Lourenço 1996	6
Heteroscorpionidae	*Heteroscorpion kaii*	Lourenço, Goodman 2009	40
Heteroscorpionidae	*Heteroscorpion kraepelini*	Lourenço, Goodman 2006	41
Heteroscorpionidae	*Heteroscorpion magnus*	Lourenço, Goodman 2002	42
Heteroscorpionidae	*Heteroscorpion raselimanana*	Lourenço, Goodman 2004	43
Hormuridae	*Opisthacanthus ambanja*	Lourenço 2014	44
Hormuridae	*Opisthacanthus andohahela*	Lourenço 2014	45
Hormuridae	*Opisthacanthus antsiranana*	Lourenço 2014	44
Hormuridae	*Opisthacanthus darainensis*	Lourenço, Goodman 2006	46
Hormuridae	*Opisthacanthus lavasoa*	Lourenço, Wilmé, Waeber 2016	47
Hormuridae	*Opisthacanthus lucienneae*	Lourenço, Goodman 2006	46
Hormuridae	*Opisthacanthus maculatus*	Lourenço, Goodman 2006	46
Hormuridae	*Opisthacanthus milloti*	Lourenço, Goodman 2008	48
Hormuridae	*Opisthacanthus pauliani*	Lourenço, Goodman 2008	48
Hormuridae	*Opisthacanthus piceus*	Lourenço, Goodman 2006	46
Hormuridae	*Palaeocheloctonus pauliani*	Lourenço 1996	6
Hormuridae	*Palaeocheloctonus septentrionalis*	Lourenço, Wilmé 2015	49

1. Lourenço WR. New taxonomic considerations on some species of the genus *Grosphus* Simon, with description of a new species (Scorpiones, Buthidae). Revue Suisse de Zoologie. 2003;110(1):141–154.
2. Lourenço WR, Goodman SM. Description of a new species of Grosphus Simon (Scorpiones, Buthidae), from the Ankarana Massif, Madagascar. Revista Ibérica de Aracnología. 2003;7:19–28.

3. Lourenço WR. A new species of *Grosphus* Simon, 1880 (Scorpiones: Buthidae) from the Southwest of Madagascar. Entomologische Mitteilungen aus dem Zoologischen Museum Hamburg. 2012;16(188):33–40.

4. Lourenço WR, Goodman SM, Ramilijaona O. Three new species of *Grosphus* Simon from Madagascar (Scorpiones, Buthidae). Revista Ibérica de Aracnología. 2004;9:225–234.

5. Lourenço WR, Wilmé L. Three new species of *Grosphus* Simon, 1880, (Scorpiones: Buthidae) from Madagascar; possible vicariant cases within the *Grosphus bistriatus* group of species. Madagascar Conservation & Development. 2016;11(2):52–65.

6. Lourenço WR. Scorpions (Chelicerata, Scorpiones). Faune de Madagascar N. 87. Muséum national d'Histoire naturelle, Paris. 1996;106pp.

7. Lourenço WR, Wilmé L, Waeber PO. One more vicariant new species of *Grosphus* Simon, 1880, (Scorpiones: Buthidae) from Madagascar. Revista Ibérica de Aracnología. 2016;29:45–50.

8. Lourenço WR, Goodman SM. Further considerations regarding the status of *Grosphus madagascariensis* (Gervais) and *Grosphus hirtus* Kraepelin, and description of a new species (Scorpiones, Buthidae). Revue Suisse de Zoologie. 2006;113(2):247–261.

9. Lourenço WR. Another new species of *Grosphus* Simon (Scorpiones, Buthidae) for Madagascar. Revue Suisse de Zoologie. 2001;108(3):455–461.

10. Lourenço WR. A new species of *Grosphus* Simon (Scorpiones, Buthidae), the first record of an intertidal scorpion from Madagascar. Entomologische Mitteilungen aus dem Zoologischen Museum Hamburg. 1999;12:297–307.

11. Lourenço WR. The genus *Grosphus* Simon, 1880 in South-Western Madagascar, with the description of a new species (Scorpiones, Buthidae). Zoosystema. 2014;36(3):631–645.

12. Lourenço WR, Wilmé L. Scorpions collected in the Makay Mountain range, Madagascar (Scorpiones: Hormuridae: Buthidae) and description of one new species. Revista Ibérica de Aracnología. 2015;26:55–61.

13. Lourenço WR. Scorpions from Mandena east coastal rain forest in Madagascar, and description of a new species of *Grosphus* Simon (Scorpiones, Buthidae). Boletín Sociedad Entomológica Aragonesa. 2005;37:83–87.

14. Lourenço WR. Scorpions du Sud-ouest de Madagascar et en particulier de la forêt de Mikea. In A.P. Raselimanana, S.M. Goodman (Eds.) Inventaire floristique et faunistique de la forêt de Mikea : Paysage écologique et diversité biologique d'une préoccupation majeure pour la conservation. Recherches pour le Développement. Série Sciences Biologiques. Centre d'Information et de Documentation Scientifique et Technique, Antananarivo. 2004;21:25–35.

15. Lourenço WR, Qi, J.-X., Goodman SM. Scorpions of south-western Madagascar. A new species of *Grosphus* Simon, 1880 (Scorpiones, Buthidae). Boletín Sociedad Entomológica Aragonesa. 2007;40:171–177.

16. Lourenço WR. A new species of *Grosphus* Simon, 1880 (Scorpiones, Buthidae) from Central Madagascar. Entomologische Mitteilungen aus dem Zoologischen Museum Hamburg. 2013;16(189):57–62.

17. Lourenço WR, Soarimalala V, Goodman SM. The species of *Grosphus* Simon (Scorpiones, Buthidae) distributed in the northern and eastern regions of Madagascar with the description of a new species. Malagasy Nature. 2009;2:144–153.

18. Lourenço WR, Wilmé L. Species of *Grosphus* Simon, 1880, associated to the group madagascariensis / hirtus (Scorpiones: Buthidae); description of a peculiar new species from the humid eastern forests of Madagascar. Entomologische Mitteilungen aus dem Zoologischen Museum Hamburg. 2015;17(194):207–223.

19. Lourenço WR, Waeber PO, Wilmé L. More about the geographical distribution of the Malagasy genus *Neogrosphus* Lourenço, 1995 (Scorpiones: Buthidae) and description of a vicariant new species. Comptes Rendus Biologies. 2015;338(11):768–776.

20. Lourenço WR. Description de trois nouveaux genres et quatre nouvelles espèces de Scorpions Buthidae de Madagascar. Bulletin du Muséum National d'Histoire Naturelle, Paris. 1995;4e série 17(1–2):95–106.

21. Lourenço WR, Ythier E. Another new species of *Pseudouroplectes* Lourenço, 1995 from Madagascar (Scorpiones, Buthidae). ZooKeys. 2010;48:1–9.

22. Lourenço WR, Goodman SM. A reappraisal of the geographical distribution of the genus *Pseudouroplectes* Lourenço (Scorpiones: Buthidae) in Madagascar. Comptes Rendus Biologies. 2006;329(1):117–123.

23. Lourenço WR, Goodman SM. Taxonomic and ecological observations on the scorpions collected in the Réserve Naturelle Intégrale d'Andohahela, Madagascar. In S.M. Goodman (Ed.). A floral and faunal inventory of the Réserve Naturelle Intégrale d'Andohahela, Madagascar with particular reference to elevational variation. Fieldiana: Zoology. 1999;new series 94:149–153.

24. Lourenço WR, Wilmé L, Waeber PO. More about the geographical pattern of distribution of the genus *Pseudouroplectes* Lourenço, 1995 (Scorpiones: Buthidae) from Madagascar. Comptes Rendus Biologies. 2016;339(1):37–43.

25. Lourenço WR, Goodman SM. A new species of *Tityobuthus* (Pocock) from Namoroka in the province of Mahajanga (Scorpiones, Buthidae). Revista Ibérica de Aracnología. 2004;9:19–22.

26. Lourenço WR, Qi J-X, Goodman SM. The identity of *Tityobuthus baroni* (Pocock, 1890) (Scorpiones, Buthidae) and description of three new species from Madagascar. Boletín Sociedad Entomológica Aragonesa. 2008;42:89–102.

27. Lourenço WR, Goodman SM. New considerations on the genus *Tityobuthus* Pocock (Scorpiones, Buthidae), and description of a new species from the Ankarana in Northern Madagascar. Revista Ibérica de Aracnología. 2003;8:13–22.

28. Lourenço WR. Another new species of *Tityobuthus* from Madagascar (Scorpiones, Buthidae). Entomologische Mitteilungen aus dem Zoologischen Museum Hamburg. 1997;12(155):147–151.

29. Lourenço WR. More about the Buthoidea of Madagascar, with special references to the genus *Tityobuthus* Pocock (Scorpiones, Buthidae). Revue Suisse de Zoologie. 2000;107(4):721–736.

30. Lourenço WR, Goodman SM. Taxonomic and ecological observations on the scorpions collected in the Forest of Ankazomivady-Ambositra and on the "RS d'Ivohibe", Madagascar. Revista de Biología Tropical. 1999;47(3):475–482.

31. Lourenço WR. A new species of *Tityobuthus* from Madagascar (Scorpiones, Buthidae). Bolletino del Museo Regionale di Scienze Naturali, Torino. 1996;14:267–273.

32. Lourenço WR, Wilmé L, Waeber PO. The geographical pattern of distribution of the genus *Tityobuthus* Pocock, 1890, a typical Ananterinae element endemic to Madagascar (Scorpiones: Buthidae). Comptes Rendus Biologies. 2016;339(9–10):427–436.

33. Lourenço WR. Further considerations regarding *Tityobuthus baroni* (Pocock, 1890) with the description of a new species from Ste Marie Island, Madagascar (Scorpiones, Buthidae). Zoosystema. 2004;26(3):385–392.

34. Lourenço WR. Un nouveau genre de scorpion malgache, maillon possible entre les Microcharmidae et les Buthidae. Comptes rendus de l'Académie des Sciences, Paris, Sciences de la Vie. 2000;323(10):877–881.

35. Lourenço WR. Humicolous microcharmid scorpions: a new genus and species from Madagascar. Comptes Rendus Biologies. 2004;327(1):77–83.

36. Lourenço WR, Goodman SM, Fisher, BL. A reappraisal of the geographical distribution of the endemic family Microcharmidae Lourenço (Scorpiones) in Madagascar and description of eight new species and subspecies. Proceedings of the California Academy of Sciences, Fourth Series. 2006;57(26):751–783.

37. Lourenço WR. Description of a new species of scorpion from the Réserve Spéciale d'Anjanaharibe-Sud, Madagascar. In S.M. Goodman (Ed.). A floral and faunal inventory of the Réserve Spéciale d'Anjanaharibe-Sud, Madagascar: with reference to elevational variation. Fieldiana: Zoology, new series. 1998;90:69–72.

38. Lourenço WR. *Microcharmus hauseri*, nouvelle espèce de Scorpion de Madagascar (Scorpiones, Buthidae). Revue Suisse de Zoologie. 1996;103:319–322.

39. Lourenço WR. Un modèle de distribution géographique présenté par les scorpions du genre *Microcharmus* Lourenço, avec la description d'une nouvelle espèce. Pattern of geographical distribution presented by the scorpions of the genus *Microcharmus* Lourenço, with a description of a new species. Comptes rendus de l'Académie des Sciences, Paris, Sciences de la Vie. 1999;322(10):843–846.

40. Lourenço WR, Goodman SM. Description of a new species of *Heteroscorpion* Birula, 1903 (Scorpiones, Heteroscorpionidae) from 'Grande avasoa' in extreme southern Madagascar. Entomologische Mitteilungen aus dem Zoologischen Museum Hamburg. 2009;15(181):115–125.

41. Lourenço WR, Goodman SM. Description of a new species of *Heteroscorpion* Birula, 1903 (Scorpiones, Heteroscorpionidae) from the Montagne des Français in extreme northern Madagascar. Zootaxa. 2006;1269:31–41.

42. Lourenço WR, Goodman SM. Scorpions from the Daraina region of northeastern Madagascar, with special reference to the family Heteroscorpionidae Kraepelin, 1905. Revista Ibérica de Aracnología. 2002;6:53–68.

43. Lourenço WR, Goodman SM. Description of a new species of *Heteroscorpion* Birula (Scorpiones, Heteroscorpionidae) from the eastern lowland humid forest of south-eastern Madagascar. Revista Ibérica de Aracnología. 2004;9:319–323.

44. Lourenço WR. Micro-endemic and vicariant populations of *Opisthacanthus* Peters, 1861 (Scorpiones: Hormuridae) in Madagascar, with descriptions of two new species. Arthropoda Selecta. 2014;23(4):383–391.

45. Lourenço WR. A new species of *Opisthacanthus* Peters, 1861 (Scorpiones: Hormuridae) from the Parc National d'Andohahela, Madagascar. Entomologische Mitteilungen aus dem Zoologischen Museum Hamburg. 2014;17(193):179–190.

46. Lourenço WR, Goodman SM. A reappraisal of the geographical distribution of the genus *Opisthacanthus* Peters, 1861 (Scorpiones: Liochelidae) in Madagascar, including the description of four new species. Boletín Sociedad Entomológica Aragonesa. 2006;38:11–23.

47. Lourenço WR, Wilmé L, Waeber PO. One more new species of *Opisthacanthus* Peters, 1861 (Scorpiones: Hormuridae) from the Lavasoa Forest, Southeast Madagascar. Revista Ibérica de Aracnología. 2016;29: 9–17.

48. Lourenço WR, Goodman SM. Scorpions of the Réserve spéciale d’Ankarana, Madagascar, with particular reference to cave-dwelling animals and the description of two new species (Arachnida, Scorpiones). Zoosystema. 2008;30(3): 665–679.

49. Lourenço WR, Wilmé L. Micro-endemic populations of *Palaeocheloctonus* Lourenço, 1996 (Scorpiones: Hormuridae) in Madagascar: A new case of vicariance among Malagasy scorpions. Arthropoda Selecta. 2015;24(2): 189–195.
