# Supplementary material for: On specimen killing in the era of conservation crisis – A quantitative case for modernizing taxonomy and biodiversity inventories
Source: PLoS One. 2017 Sep 13;12(9):e0183903. doi: 10.1371/journal.pone.0183903 (PMC5597100; doi:10.1371/journal.pone.0183903)
Supplement: S3 Table — (The MZFAA took place from April 1929 to May 1931). (PDF) [file pone.0183903.s005.pdf]

S3 Table. The taxa in the new Bernieridae endemic family and the number of birds killed which are documented in the Noe4D database. (The MZFAA took place from April 1929 to May 1931).

**Family Bernieridae**	**Number of birds killed and documented in Noe4D**		
	**IV 1929 – V 1931**	**1990 – 2010**	**Total (1867 – 2010)**
Cryptosylvicola randrianasoloi Goodman et al. 1995	0	11	11
Hartertula flavoviridis Hartert 1924	12	13	41
Oxylabes madagascariensis (Brisson 1760)	45	60	139
Thamnornis chloropetoides (A. Grandidier 1867)	20	8	33
Crossleyia xanthophrys (Sharpe 1875)	4	13	44
Bernieria madagascariensis (Gmelin 1789)	4	6	44
Bernieria m. madagascariensis (Gmelin 1789)	54	74	177
Bernieria madagascariensis inceleber Bangs, Peters 1926	58	38	102
Xanthomixis zosterops (Sharpe 1875)	1	26	64
Xanthomixis z. Zosterops (Sharpe 1875)	49	26	103
Xanthomixis zosterops fulvescens (Delacour 1931)	14	0	17
Xanthomixis zosterops andapae (Salomonsen 1934)	11	20	31
Xanthomixis zosterops ankafanae (Salomonsen 1934)	0	33	33
Xanthomixis apperti (Colston 1972)	0	12	12
Xanthomixis tenebrosa (Stresemann 1925)	2	1	21
Xanthomixis cinereiceps (Sharpe 1881)	10	55	93
Randia pseudozosterops Delacour, Berlioz 1931	5	0	5
**Totals**	**289**	**396**	**970**
